# Robust design of a logistics system using FePIA procedure and analysis of trade-offs between CO_2_ emissions and net present value

**DOI:** 10.1016/j.heliyon.2023.e18444

**Published:** 2023-07-25

**Authors:** Andrés Polo Roa, John Willmer Escobar, María Paula Montoya

**Affiliations:** a Departament of Industrial Engineering, Fundación Universitaria Agraria de Colombia, Bogotá 110110, Cundinamarca, Colombia; b School of Industrial Engineering, Universidad del Valle, Cali, Cali 760001, Valle del Cauca, Colombia; c Department of Accounting and Finance, Universidad del Valle, Cali, Cali 760001, Valle del Cauca, Colombia

**Keywords:** Sustainability, Decision support system (DSS), Supply chain, FePIA

## Abstract

The problems of flexible planning of the design of logistics systems for the collection of food products such as raw milk can result in a decrease in the performance of critical indicators for their performance. This paper proposes a new efficient methodology for robustly designing a first-mile logistics system for storing and refrigerating milk as a perishable product considering decisions related to open facilities and the flow of products, including sustainability indices. The proposed approach is modeled as a bi-objective problem by considering the minimization of greenhouse gas emissions (CO2) produced by milk transportation canteens and the maximization of the system configuration's net present value (NPV). We have analyzed and determined the most robust configuration for the first time and explained the robustness-NPV and robustness-CO2 relationships. The proposed mathematical model is solved by the Epsilon constraints method, and the robustness is calculated considering an extension of the FePIA methodology for multiobjective problems. A novel contribution is a balance in the possible future values generated by the company related to its cash flows and the generation of CO2 emissions when using a motorized transport frequently used in the shipment of raw milk considering a new important aspect such as the volume of product transported and the slope of the path between the production farm and the storage cooling tanks.

## 1 Introduction

Adopting modern technologies is crucial to improving the productivity and well-being of poor farmers in developing countries [1]. Studies have indicated that value chains are essential in adopting technology by processing companies and farmers through vertical coordination [2]. One of the factors that can drive growth in the primary sector of emerging economies is the demand for milk and dairy products, which has been growing in recent years [3]. This market's growth source is to guarantee quality and safety throughout the supply chain. In the dairy industry, quality and safety issues occur more frequently upstream of the supply chain [4], forgetting the importance of first-mile logistics for this chain ( Graph 2, Graph 3, Graph 4, Graph 5, Graph 6).

In 2021, the value of the dairy market worldwide was estimated to be about 871 billion U.S. dollars, projected to grow to 1,128 billion dollars by 2026 ([5]). Milk production in Colombia has increased steadily, with an average growth rate of 3.5% during the last twenty years ([6]). The challenges facing the dairy sector make it necessary to generate the need for strategic planning as a vitally important foundation for the study not only at a sectoral level but also at a business and social level ([7,8]). However, given the limited knowledge of decision support methodologies, small and medium-sized companies' possibilities of quality, safety, competitiveness, productivity, and innovation are affected ([9,10]). Besides, the life cycle of livestock systems is not assessed, evaluating their environmental performance ([11]). The income received by primary producers is a topical issue with uncertainty about the short-term dairy sector in Colombia. The crisis in the sector occurs during a susceptible situation due to the foreign trade policy that the National Government has drawn up for more than ten years. The evident growth of imports from the United States and Mercosur, which are beginning to serve the Colombian market and local production, has generated an oversupply that makes the dairy business unfeasible. Given this scenario, primary producers sought to be more efficient in production costs, forgetting the costs associated with the distribution of raw milk. According to data from the Colombian Association of Milk Processors in 2022, an 8.3% recovery of formal milk collection was observed, translating into a formality growth in primary production SMEs. Thus, one of the main challenges for the future of small primary producers is to maintain a competitive and profitable business within an uncertain economic and political context ([12]), and a fundamental part of this challenge is based on the sustainable design of the collection system and distribution of raw milk.

The design of a production system is recognized as a critical factor in achieving competitive advantages since its management allows the systematic integration of innovation processes and the strengthening of business strategy ([9,13]). If this design is accompanied by investments in information technology combined with research and development, it is possible to improve the performance of small businesses in Colombia ([14]). A challenge in designing a logistics system for collecting raw milk is the existence of several uncertainties related to demand ([15,16]), with the coordination between a dairy company and its multiple suppliers of raw milk ([4,17]), and with prices and costs ([18,19]). Therefore, considering deterministic assumptions for parameters of milk supply chain problems could result in infeasible or suboptimal solutions ([4,[20], [21], [22]]).

One more challenge is achieving a logistics system's sustainability ([6,[23], [24], [25]]). Among the aspects that should be considered are logistics operations' environmental and social impacts, such as transportation and the financial and accounting impacts on companies ([26,27]). A sustainable logistics system design for the dairy industry has become a topic of considerable interest in the literature ([[28], [29], [30], [31]]). Therefore, striking a proper balance between the different conflicting dimensions of sustainability - economic, environmental, and social objectives - is an issue that must be addressed to design a sustainable milk collection logistics system robustly. Likewise, sustainability indicators are helpful for decision-makers in the design of a supply chain ([32]).

One way to address the inherent uncertainty in logistics systems is by considering the concept of robustness. The paper proposed in Ref. [33] defines a *robust solution* to a problem as an immune strategy to manage the uncertainty of the data. Besides [34], defines *robustness* as the preservation of specific desired characteristics of the system, despite fluctuations in the behavior of its parts or the environment. Thus, a system is robust if it can maintain its performance characteristics at an acceptable level in the face of unexpected and uncertain variations of parameters initially assumed to be invariable ([35]).

Although many publications are available in the logistics system modeling field, some gaps still need to be covered. Mathematical models addressing the robust design of sustainable logistics systems, especially in collecting and processing raw milk, are limited. Based on the above, the following research gaps require further attention:

• The strategic design of the raw milk collection system deals with the following logistics operations decisions: (i) location of cooling tanks; (ii) assignment of primary producers to each cooling tank; and (iii) transportation plans for the distribution of the raw milk collected. Therefore, the proposed methodology addresses collected raw milk's location and distribution decisions as an integrated problem.
• The robust design of the sustainable logistics system for collecting and processing raw milk requires a multi-objective analysis and various robustness requirements based on social, environmental, and economic aspects.
• The performance of the sustainable logistics system should be based on financial, environmental, and social aspects providing a more realistic analysis to support decision-making. The composition of decisions of location (strategic), use of modes of transportation, use of cooling tank capacities (tactical), and distribution (operational) within a supply chain problem of several periods must guarantee a better performance of the global system.
• Emissions are among the best-known and most-used environmental impact indicators. This paper broadens the use of sustainability indicators based on the sustainable development goals (SDG), such as Health and well-being (SDG 3); Decent Work and Economic Growth (SDG 8); Industry, Innovation, and Infrastructure (SDG 9); Sustainable communities (SDG 11); Responsible Consumption and Production (SDG 12); Climate Action (SDG 13) and Peace, Justice and Strong Institutions (SDG 16).
• As the first echelon in the dairy supply chain belonging to the agri-food industry, the milk collection logistics system must also face climate problems. This industry is expanding and needs to be efficiently designed to meet the demand for this food, taking care of its natural ecosystem.
• The raw milk collection system's design must consider the study area's geographical features to generate solutions.

Based on the design of a logistics network, this research seeks to answer the following research questions (RQ):

• RQ1: How can a first-mile logistics network for raw milk collection be modeled based on its dynamic processes?
• RQ2a: How is the behavior of the performance characteristics of the logistics system when it is disturbed by unforeseen events?
• RQ2b: What is the robust design that mitigates the effects of these disturbances?

This paper offers a practical contribution to the improvement of small producers on investments in environmentally sustainable raw milk collection and distribution systems for the management of transportation modes, especially by using motorcycles on slopes, also improving social aspects related to human effort. These aspects typify the triple-bottom-line perspective of sustainability, capturing a broader social and environmental point of view rather than just considering the economic issues of primary raw milk production.

The development of research on the robust design of a sustainable logistics system for collecting raw milk considering efficiently a variety of future scenarios, which involve decision-making in line with strategic planning under uncertainty, is essential. This paper proposes a collaborative framework for decision-making for the sustainable logistics system design for the first mile of raw milk transport. This framework facilitates the development of collaborative relationships in a network to improve the sustainability of delivered products.

This paper proposes a bi-objective mathematical model and a robustness measurement scheme covering the identified gaps in supply chain topics. The first objective function seeks to maximize the Net Present Value of the money flows obtained by the milk collection logistics system in various periods. The second objective function seeks to minimize the CO_2_ emissions produced by motorized transport frequently used in the shipment of raw milk to cooling tanks, taking into account the volume of product transported and the slope of the path between the production farm and the storage cooling tank. The proposed scheme considers collaboration and sustainability functionalities remaining to be evaluated in the objective functions. Consider various decisions about the location-allocation of cooling tanks, used capacity of tanks, and net profits obtained by each company associate, among others.

The main contributions of this paper focus on several aspects. First, the paper extends and customizes the proposed robustness methodology by Refs. [35,36] for supply chain design considering several objectives. Therefore, multi-objective mixed integer programming (PEMMO) approaches the logistics network design problem, and robustness measurement through the FePIA procedure for multiple objectives is considered (maximization of the Net Present Value (NPV) and the minimization of environmental effects, considering fuel consumption). Second, this paper proposes a new transportation network design scheme, considering minimizing fuel consumption. The fuel consumption depends mainly on the distance and the load, considering the forces of the vehicle to transport products on sloping terrain. Finally, the paper has considered a study of a real problem related to the design of logistics systems for which new constraints focused on maintaining the connectivity requirements of the problem are incorporated. The obtained results demonstrate the applicability of this study and the implementation of the model in ambiguous and uncertain conditions.

Additionally, this paper presents an exhaustive review of the literature that supports the design of the mathematical model and the methodology used for the study. The proposed methodology can be extended to any company in the sector with similar characteristics.

## 2 Literature review

The literature review is based on related papers considering the importance of designing robust logistics systems including various objective functions, especially those focused on sustainable logistics systems.

### 2.1 Supply chain robustness

Regarding the design of robust logistics systems, problems in recent years have been addressed chiefly with methodologies based on robust optimization. Robust optimization manages the uncertainties involved in optimization models ([37]). Although stochastic approaches are impractical within uncertain environments where the distribution function of the uncertain parameters is unknown, for [38], robust optimization methods are reasonably practical because these parameters can be modeled continuously or as discrete scenarios.

Some authors used the approach developed considering linear optimization problems ([[39], [40], [41], [42]]). In particular, the solution is guaranteed to remain feasible and best when the information changes. Other authors developed their algorithms when it was impossible to use previous approaches ([43,44]). These approaches work under the information probability approach, and the concept of robustness is specifically mathematical. Some authors proposed robust models based on Fuzzy Sets. Works such as those by Refs. [[45], [46], [47]] addressed parameter uncertainty through fuzzy programming by guaranteeing the "flexibility" of the operators, especially constraints, or the "possibility" of the distribution of uncertain parameters.

Other recent approaches were developed to address uncertainty in logistics system design problems by incorporating robustness metrics. Authors such as [48,49] proposed an integrated modeling framework to study cascading faults and evaluate the robustness of logistics networks with the simile of electrical systems. This approach was used for generic supply chains, but these concepts have yet to be applied to specific cases. The authors examined the fraction of failed entities under load decay and fluctuation scenarios. An approach developed by Ref. [36] based on [34], considers the ability of the logistics system to preserve its specified properties against expected or unexpected disturbances. In particular, the approach quantitatively determines the robustness of the system.

Robust possibilistic programming approaches for supply chain design have been hardly studied by Refs. [[50], [51], [52], [53], [54], [55]]. In the work [50], the authors introduce a s biogas supply chain network design model considering the triple bottom line approach. A hybrid strategy combining flexible and possibilistic programming has been proposed to solve problem. A fuzzy possibilistic approach for solving a biomass-wood pellet supply chain has been studied by Ref. [51]. The objective function is minimizing the total supply chain cost by reducing carbon emissions. The work [52] proposes a multiobjective mathematical model considering the minimization of the total CO2 emissions and the total cost of the supply network. A robust possibilistic approach has been proposed to handle existing inaccurate data. In Ref. [53], a blood supply chain network within a disaster environment is studied. A robust possibilistic multiobjective optimization model is proposed considering the minimization of the three objective functions: the total cost, the waiting patient cost, and the unfulfilled demand. A combining methodology combining a possibilistic programming model and simulation approach for an organ supply chain network has been proposed by Ref. [54]. Finally, the authors of [55] proposed a robust possibilistic approach to solve a multiobjective model of a closed supply chain, minimizing the carbon emissions and the total cost and maximizing the system's responsiveness.

Multi-objective robust supply chain design problems have been studied hard by several authors ([[56], [57], [58]]). A multi-objective robust approach for a sustainable supply chain, maximizing an equitable distribution of the supplies and minimizing the operating costs based on transportation times, has been proposed by Ref. [56]. A multi-objective model for a pharmaceutical supply chain network considering several echelons, products, periods, and transportation modes has been introduced [57]. In Ref. [58], a multi-objective location inventory routing model for a blood supply chain has been proposed [58]. Strategic operational and tactical decisions have been considered a disruption of the network and blood shelf-life.

Kaoud et al. [59] consider a robust multi-objective model for a green closed-loop supply chain network considering maximizing the profit and minimizing the carbon emissions. This proposed model considers presorting, heterogeneous fleet, and uncertainty on costs and selling prices. Al-Ashhab [60] propose a multi-objective mode for a closed-loop supply chain network maximizing the total profit of the network and the customer service level and minimizing the total cost. Saffari et al. [61] propose a horizontal collaboration approach for a resilient and sustainable logistic network considering costs, social responsibility, CO2 emission, water consumption, and responsiveness time. A multi-objective mathematical model is proposed to solve the considered problem. Rabbani et al. [62] propose a stochastic multi-objective model for a phosphorus supply chain network considering different products and periods.

Shiri et al. [63] propose a multi-objective model for a home healthcare supply chain considering two stages. The first stage considers strategic decisions related to open facilities, and the second stage considers tactical and operational decisions such as routing and scheduling. The objective functions consider minimizing the total cost and maximizing social aspects. Finally [64], considers a closed-loop supply chain design problem by considering financial criteria such as maximizing the net present value (NPV) and minimizing the financial risk (FR).

Table 1 summarizes the central literature review, highlighting that the robust system design approach deserves more research attention.

**Table 1:** Main papers robust optimization system logistics literature review.

Authors	Robust optimization	Robust design	Objective Functions	Solution Method	Type Supply Chain
Shu et al. [65]		X	Robustness of supply chains	Firefly algorithm	General supply chain system
Ma et al. [94]	X		Economical Cost	Robust multi-objective	Environmental closed-loop supply chain
			Environmental cost	Mixed integer nonlinear programming model	
Aras & Bilge [67]	X		Total cost	Robust optimization	Food supply chain
Polo et al. [36]		X	Economic value-added	FePIA procedure	Closed-loop supply chain Electrical industry
Abdolazimi et al. [68]	X		Delivery time of purchased materials	Robust optimization	Closed-loop supply chain of a Tire Factory
			The network overall profitability	VIKOR method	
			Environmental impacts		
Ghaderi et al. [69]	X		Total Costs	Interactive fuzzy programming	Bioethanol supply chain
			Environmental impacts		
			Switchgrass production		
			Holding inventory		
			Social responsibility		
Hombach et al. [39]	X		Total profit	Robust optimization approach	Biodiesel Market
				ϵ-constraint method	
			Total emissions		
			Land use change		
Ghahremani-Nahr et al. [45]	X		Total cost	Robust fuzzy optimization approach	Closed-loop supply chain
				Whale optimization algorithm	
Sherafati et al. [40]	X		Total profit	Robust optimization approach	Supply chain cable industry
			Balanced regional development	ϵ-constraint method	
Mohseni and Pishvaee [41]	X		Total cost	Data-driven robust optimization	Wastewater sludge to biodiesel supply chain
Hassanpour et al. [43]	X		Collection ratios of used products under various quality types	Robust optimization approach	Closed-loop supply chain Electrical industry
				Particle swarm optimization-genetic algorithm	
Larimi & Yaghoub [70]	X		Total cost	Robust-stochastic approach whit AHP criteria	Blood supply chain
			Delivered PLT units		
Jabbarzadeh et al. [71]	X		Total cost	Robust optimization approach	Pharmaceutical supply chain
			Environmental Impacts		
Tosarkani et al. [72]	X		Total expected profit	Robust possibilistic approach	Waste Electrical and Electronic Equipment
			Environmental compliance		
Zarbakhshnia et al. [73]	X		Total cost	Robust optimization approach	Waste Electrical and Electronic Equipment
			CO_2_ emissions	ϵ-constraint method	
Wei & Dong [74]	X		Logistics cost	Adaptive-weight Genetic Algorithm	Dry-port based logistics network
			Time of unit container transportation		
Vafaeenezhad et al. [75]	X		Total profit	Augmented ε-constraint method	Supply chain pulp and paper industry
			GHG emissions		
			Total consumed energy		
			Total generated wastes		
			Total travel distance of employees		
Chalmardi & Camacho-Vallejo [76]	X		Total cost	SA-based algorithm	General supply chain system
			Environmental impact		
Resat & Unsal [77]	X		Total cost	Mixed-integer linear multi-objective mathematical model	Packaging industry
			Time passed during all processes	Analytic Hierarchy Process	
			Raw materials purchased		
Darestani & Hemmati [78]	X		Total network costs	Robust optimization with overall weighting method and Torabi-Hassini method	Closed-loop supply chain for perishable goods
			Greenhouse gas emissions		
Saedinia et al. [79]	X		Total cost	Robust optimization approach	Gasoline closed-loop supply chain
			Volume of surplus demand		
Hamdan & Diabat [80]	X		Time and cost delivering	Robust optimization approach	Blood supply chain
				Lagrangian relaxation	
Nayeri et al. [81]	X		Total cost	Robust fuzzy optimization approach	Closed-loop supply chain for a water tank
				Whale optimization algorithm	
Gholizadeh & Fazlollahtabar [42]	X		Total profit	Robust optimization	Melting industry
				Genetic algorithm	
Gilani et al. [46]	X		Total profit	Robust possibilistic programming	Bioethanol supply chain network
			Environmental impacts	Fuzzy-integrated data envelopment analysis	
Liu et al. [47]	X		Total cost	Robust optimization	Green closed-loop supply chain for food industry
				Fuzzy mean-UPM model	
Sharifnia et al. [82]	X		Customer satisfaction	Robust simulation optimization	Petrochemical Supply chain
				Artificial neuronal network (ANN) metamodel	
Achmad et al. [37]	X		Total cost	Robust optimization approach	Food supply chain
			Demand fulfilment	Agent-Based Modeling	
Yang et al. [48]		X	Cascading failure model	Node load loss propagation scheme	General supply chain system
			Efficiency of supply chains	Multi-objective Firefly Algorithm	
Shafiee et al. [44]	X		Total costs	Robust optimization approach	Dairy industry
			Environmental impacts	Augmented ε-constraint method	
			Social impacts		
Gholizadeh et al. [83]	X		Environmental impact	Robust optimization approach	Dairy industry
			Total profit	Augmented ε-constraint method	
Shi et al. [49]		X	Cascading failure model	BA Model	General supply chain system
				WS model	
Ahmadvand & Sowlati [84]	X		Total cost	Robust optimization	Biodiesel supply chain
Habib et al. [85]	X		Total cost	Robust possibilistic programming	Biomass supply chain
Hosseini Dehshiri et al. [86]	X		Total cost	Robust stochastic, possibilistic, and flexible approach	Mass-producing stone paper supply chain
			Transit time	Basic flexible programming (BFP) model	
			Carbon emissions		
Krishnan et al. [87]	X		Total cost	Robust optimization	Food supply chain
			GHG emissions		
			Jobs created		
Gilani & Sahebi [38]	X		Total cost	Data-driven robust optimization	COVID-19 vaccine supply chain
			Environmental impacts		
			Jobs created		
Golpîra & Javanmardan [88]	X		Carbon emissions	Robust optimization	Sustainable closed-loop supply chain

### 2.2 Sustainable robust design

The concept of sustainability plays a vital role in distribution supply networks ([40,89]). The influence of supply chains must be determined concerning the main dimensions of sustainability: economic, environmental impact, and social responsibility ([[76], [90], [91]]). Economic performance simultaneously considers the ability to create sustainable future profits ([92]). In the logistics system design literature, many studies have considered single objectives to evaluate the economic performance of the system, such as minimizing total costs ([[93], [94], [95]]) and maximizing total earnings ([96]). Regarding the environmental impact, the studies focused on analyzing the CO2 emissions reducing the consumption of energy obtained from fossil fuels. This situation has caused drastic changes due to growing CO2 emissions and other atmospheric pollutants ([46]). For this reason, most studies focus on designing logistics systems that minimize greenhouse gas emissions ([97,87]). There needs to be documentation on the design of logistics systems that use social responsibility optimization as the only criterion for social aspects. Some authors use social aspects in the robust design of logistics systems based on multi-objective programming ([[69], [75], [87]]).

Likewise, studies have analyzed two objective functions for the design of sustainable logistics systems ([76,87,[72], [74], [83]]). Logistics systems for food products require considerable labor and significantly impact society. For this reason, it is crucial to guarantee the well-being of the people involved in the operations of the entire system, especially those linked to the first mile. Existing studies need to evaluate social sustainability aspects in the design of the network of first-mile logistics systems. This study considers the three dimensions of sustainability for the robust design of a logistics system providing a healthy and safe environment for workers and community development. A review of the literature related to sustainable supply chain network design could be consulted in Refs. [[98], [99], [100]].

In order to balance economic issues with environmental and social disturbances in the supply chain network design, the idea of a resilient and sustainable supply chain network design offers a viable solution. In order to allocate raw materials or finished goods to suppliers, manufacturers, distributors, and retailers, a supply chain network must be strategically and tactically designed. One proposal of this work is to present a systematic and bibliometric overview of the idea and term "supply chain network design." We consider 155 papers from 2010 to 2022 assessed and categorized using the VosViewer software. Research objectives, keywords, highly referenced papers, productive journals, and attributes considered for each sustainability factor were analyzed. The visualization of bibliometric literature reviews is performed using VosViewer. Future academic and professional work on supply chain network design will benefit from the findings of this study.

Several authors have recently examined real-world applications of sustainable, reliable supply chain design. The design of a sustainable lead-acid battery supply chain network is covered by Ghalandari et al. [101]. A two-stage model has been proposed to address the issue under consideration. A multi-objective mixed integer linear programming model was created by Negarandeh and Tajdin [102] to solve the hospital waste management supply chain in the hospitals in Sari, Iran. The model aims to create a network that takes uncertainty, resilience, and sustainability. An effective goal programming method and the Lp-metric method are considered to solve the model after using a robust fuzzy programming methodology. In order to bridge the gap between blood donors and consumers, Khodaverdi et al. [103] consider a blood supply chain problem. A multi-objective mixed integer linear programming model is considered while considering the economic, environmental, and social objectives. A five-echelon blood supply chain network is shown.

Alshurideh et al. [104] investigate supply chain (SC) robustness and cyber resilience effects on supply chain performance in the UAE chemical sector. Data is gathered from factories producing chemicals in Abu Dhabi, United Arab Emirates. For data analysis, a reliable sample of 303 individuals is used. The performance of the supply chain is found to directly benefit from a substantial level of cyber resilience and SC robustness. Abdali et al. [105] take a three-stage strategy To construct a sustainable sugarcane-based bioenergy supply chain network in an uncertain environment to construct a sustainable sugarcane-based bioenergy supply chain network in an uncertain environment. In the first step, fuzzy data envelopment analysis is used to identify where sugarcane fields should be opened based on climatic, ecological, and sociological factors. The second stage proposes a resilient mixed-integer linear programming model (MILP) to maximize strategic and tactical decision factors. In the final stage, an experimental analysis is carried out. In order to determine whether the created model is applicable, a genuine case study from Iraq is used. In the Meghalaya region of the Indian Himalayas, Babu et al. [106] consider sustainable agricultural food production systems that may provide household-level food security with a minimal environmental impact.

Recently, multi-objective sustainable supply chain design problems have been studied by Refs. [107,108]. Alizadeh-Meghrazi et al. [107] proposed a robust multi-objective model to study sustainable methods for a mask supply chain network design. This model's applicability is illustrated for Canada's Greater Toronto Area. Dehshiri et al. [108] investigate the design of a closed-loop supply chain network considering financial and ecological concerns regarding returned goods. This paper suggests a novel resilient optimization-based flexible, probabilistic, and stochastic programming approach.

Table 1 summarizes the most relevant literature related to some topics of the proposed problem. Research gaps are identified through the literature review, and this work's contributions are revealed. The literature review indicates that the robustness measurement of a multi-objective sustainable logistics system has yet to be examined through robustness metrics incorporating uncertainties that affect its performance. Most works use robust optimization to determine the logistics system's design, but only some address the system's robustness using robustness metrics. In addition, research on first-mile logistics design problems for perishable products is still being conducted. However, due to the perishable nature of fresh produce, supply chain members need to explore optimal sustainable operational decisions while preventing product spoilage. This study makes contributions by filling these research gaps. When sustainable aspects are considered in the robust design of logistics systems, minimizing the total cost or maximizing the total utility is the most considered objective function for economic aspects. Operational and financial decisions need to be considered simultaneously. Additionally, studies need to consider the social dimension of sustainability when designing a first-mile logistics system for perishable products despite the operation's impact on the upstream supply chain.

There is a need to develop generic models to solve real problems related to the design of logistics systems, generating value for all those involved and emitting fewer greenhouse gases from used vehicles for transportation. This aspect is the main objective of the present work, where a generic model is intended to inform decision-makers who manage these logistics systems and who can participate in tenders to acquire funds that make their businesses more competitive. The flexible model can be extended to logistics systems with similar characteristics.

## 3 Proposed methodology

The development of this research extends the methodology described by Ref. [34] for the problem of multi-objective network design considering the scenario generation technique based on historical data. In particular, it is assumed that the historical behavior of the supply of raw milk determines the future of the milk collection system. The number of scenarios varies depending on the parameters and the uncertainty included in the proposed model.

### 3.1 Problem characteristics

This section describes the base problem for formulating the proposed robust design of a sustainable raw milk collection system. The problem seeks to decide strategic (open tanks for producer-associated) and tactical decisions (flow and inventory of products) related to the supply chain design of raw milk. The network considers several periods, a single product, and a network of farmer producers. The milk supply chain involves different farmers and raw milk collection tanks, which could be located in the same locations as the producers to facilitate handling and maintenance. Thus, the system network consists of a single type of node. Any producer whose property does not have a cooling tank must transport the milk to an assigned tank in two modes: a motorcycle with a wheelbarrow for transporting canteens or on foot. As there are producers associated with tanks, these groups are known as a "cluster" in this work.

The proposed model contains two types of binary variables. One variable determines whether a cluster is created, while the other specifies whether a producer belongs to a created cluster, including whether the cooling tank is installed on their property. The model seeks to maximize the Net Present Value (NPV) of all money flows in various periods and minimize CO_2_ emissions from motorcycles. Each node was assigned a number to label each producer, and the coordinates were collected in situ using a global positioning system (GPS) for the case study. Additionally, the use of slopes for transporting raw milk on motorcycles was taken into account in such a way that the work carried out by the engine is considered for calculating CO_2_ emissions.

### 3.2 Proposed mathematical model

#### 3.2.1 Assumptions and characteristics

The proposed model considers the following assumptions:

• A single product is considered.
• The model considers the flow of the only product in the network forward, considering strategic and tactical decisions.
• Cooling tank storage capacity constraints are considered. It is assumed that a cooling tank could be located in any producer location.
• Tanks have a limited capacity.
• There are multiple periods for making decisions.
• The locations of the farmer producers are specified.
• All milk supply from suppliers must be transported to some tank.
• Producers can only be linked to a single cluster.
• Scarcity is not allowed.
• All costs considered are deterministic and known a priori.

The problem is of great interest because the location of a specific tank implies the opening of facilities. This situation forces dairy companies to seek design or redesign strategies for their logistics systems based on optimization tools to maintain high competitiveness and seek sustainable development. In this paper, the model considers the number of cooling tanks to locate, their capacity, and the location, which raises the following questions: How many tanks should be used? Where is it convenient to open the tanks? Is an expansion (open more tanks) or contraction (remove tanks) convenient? Is it possible to find an optimal configuration of the milk collection logistics system considering the robustness of changes in raw milk supply and its selling prices?

The following indices, parameters, and variables were defined to solve the problem:

#### 3.2.2 Sets

i Producers i=1,2,…,I.

m Clusters m=1,2,…,M.

t Type of storage tank t=1,2,…,T.

j Periods j=0,1,2,…,J.

#### 3.2.3 Parameters

PMAX: Maximum annual production of milk per bovine (lts/year)

PMIN: Minimum annual production of milk per bovine (lts/year)

PREMAX: Maximum raw milk selling price (COP/lts)

PREMIN: Minimum raw milk selling price (COP/lts)

${NBOV}_{i}$: Number of bovines of the producer i (number of bovines/producer)

${Ofer}_{ij}$: Average annual supply of milk from producer i at period j (lts/year)

${Prec}_{j}$: Selling price of milk for each year j (COP/lts)

${O\_tot}_{j}$: Total milk supply for each year j (lts)

${CANT}_{ij}$: Number of transported canteens by each producer i for each year j (canteens/year)

${AH}_{i}$: Producer Abscissa i

${OH}_{i}$: Producer ordinate i

$X_{m}$: Abscissa of the location of the cooling tank corresponding to cluster m

$Y_{m}$: Ordinate of the location of the cooling tank corresponding to cluster m

$D_{im}$: Linear distance between the producer i and the location of the cooling tank m (km)

${vinf}_{t}$: Infrastructure value for tank t (COP/facility)

${val}_{t}$: Capacity tank value t (COP/tank)

${Capt}_{t}$: Capacity tank t (lts/year)

${CA}_{tj}$: Depreciation cost of tank t during time period j (COP/tank x year)

${CIN}_{tj}$: Infrastructure depreciation cost of tank t in period j (COP/facility x year)

${CRF}_{tj}$: Cost of cooling milk in a cooling tank of capacity t in period j (lts/tank x year)

$\beta_{im}$: Angle of inclination of the distance between producer i and cluster m (radians)

${BBIN}_{im}$: Binary parameter of the angle of inclination of the distance between producer i and cluster m to verify the direction of the angle. 1 if the angle is up, 0 if it is down

${Inter}_{j}$: Credit interest rate for infrastructure adjustments in period j (COP/year)

CTra: Transportation cost of milk on foot to the tank (COP/km)

TrMot: Transportation cost of milk by using motorcycle (COP/km)

Sue: Salary of workers (COP/year)

Eco: Value of computer equipment (COP/equipment)

Eof: Office equipment value (COP/equipment)

Vac: Purchase value per bovine (COP/Bovine)

Maq: Equipment value (COP/equipment)

Ces: Consolidated unemployment pay (COP/year)

IntCes: Unemployment interest year (COP/year)

Vcion: Consolidated vacations (COP/year)

Ir: Discount rate

PMot: Weight of the motorcycle including the rider (Kg/motorcycle)

Pcar: Empty weight of the cart (equipment to carry out on foot) (kg/Cart)

PLec: Weight of a liter of milk in kg

Pcant: Weight of an empty milk canteen in kg

Gra: Gravity (m/s2)

Coef: Tire-ground friction coefficient

Ace: Acceleration (m/s2)

E1: Work conversion factor (W) to (gallons/joules)

E2: Conversion factor of the amount of emission per unit of fuel (Kg of CO2/gallon)

Frair: Air friction (N)

${Ptra}_{i,j}$: Weight transported per producer per year (kilograms/year)

${Moto}_{i}$: Binary parameter if producer i transports milk by motorcycle

${Feet}_{i}$: Binary parameter if producer i transports the milk on foot

PVT: Percentage of total milk sales (%)

PCC: Purchase percentage of concentrate (%)

PCF: Fertilizer purchase percentage (%)

PVI: Inventory value percentage (%)

PGV: Percentage of miscellaneous expenses (%)

PGA: Percentage of administration expenses (%)

PIMP: Tax rate (%)

PIF: Ending inventory calculation percentage (%)

PCT: Calculation percentage of tank capacity (%)

${VenP}_{ij}$: Milk sales of each producer i for each period j (COP)

${VenT}_{j}$: Total sales of milk for each period j (COP)

${Conc}_{i,j}$: Purchases of bovine’s concentrate for each producer i at each period j (COP/year)

${VCon}_{j}$: Total purchase value of concentrate for each period j (COP/year)

${Fert}_{ij}$: Purchases of fertilizer for planting grass in each producer i in each period j (COP/year)

${VFert}_{j}$: Total purchase value of fertilizer in each period j (COP/year)

${InvPr}_{ij}$: Value of inventories by producer i in period j (COP/year)

${InvFin}_{ij}$: Final inventory value of producer i in period j (COP/year)

${Inv}_{j}$: Initial inventory value in period j (COP/year)

${InFin}_{j}$: Final inventory value in period j (COP/year)

${Obligac}_{j}$: Liabilities in period j (COP/year)

${PObligac}_{j}$: Debt liabilities in period j (COP/year)

${ObligFinanCP}_{j}$: Debt in the period j (COP/year)

${PObligFinanCP:}_{j}$: Debt payment in the period j (COP/year)

${CCARG}_{imj}$: Parameter representing the producer i belonging to cluster m on a motorcycle with load for period j (kilograms)

${SCARG}_{imj}$: Parameter representing the producer i belonging to cluster m on a motorcycle without load for period j (kilograms)

ti: Depreciation Capital Fraction

${W1}_{imt,}$: Motorcycle engine work climbing with load

${W2}_{imt,}$: Motorcycle engine work climbing without load

${W3}_{imt,}$: Engine work the motorcycle descending with load

${W4}_{imt,}$: Engine work the motorcycle descending without load

3.2.4Variables

*CO*_*2*_: Total emissions (kilogram/km)

*VPN:* Net Present Value (COP)

${NetProfit}_{j}$: Net profit for period j (COP/year)

${OperatProfit}_{j}\text{:}$: Operational profit for period j (COP/year)

${GrossProfit}_{j}\text{:}$: Gross profit for period j (COP/year)

${CTransp}_{j}$: Transportation costs for period j (COP/year)

${CVentas}_{j}\text{:}$: Cost of sales for period j (COP/year)

${PGAdmon}_{j}\text{:}$: Payment of administration expenses for period j (COP/year)

${PImp}_{j}$: Taxes for period j (COP/year)

${PGVarios}_{j}$: Payment of miscellaneous expenses for period j (COP/year)

${GVarios}_{j}$: Miscellaneous expenses for period j (COP/year)

${Obligac}_{j}\text{:}$: Liabilities for period j (COP/year)

${PObligac}_{j}\text{:}$: Payment of liabilities for period j (COP/year)

${ObligFinanCP}_{j}\text{:}$: Financial debts for period j (COP/year)

${PObligFinanCP}_{j}\text{:}$: Payment of financial debts for period j (COP/year)

${GAdmon}_{j}\text{:}$: Administration expenses for period j (COP/year)

${GInteres}_{j}$: Interest expenses for period j (COP/year)

${Taxes}_{j}$: Taxes for period j (COP/año)

${InvIn}_{j}$: Initial investment of company

${FEA}_{j}\text{:}$: Annual cash flow for period j (COP/year)

${FE}_{j}\text{:}$: Cash flow for period j (COP/year)

${CVProd}_{ij}\text{:}$: Cost of sales produced by each producer i for period j (COP/year)

${CTProd}_{ij}\text{:}$: Transportation cost for each producer i at given period j (COP/year)

${ProdOperatPro}_{ij}\text{:}$: Operational profit for each producer i in period j (COP/year)

**Binary Variables**

$Z_{mt}$: 1 if the cluster m is open, 0 otherwise

$W_{mi\;t}$: 1 if producer i belongs to cluster m, 0 otherwise

#### 3.2.5 Objective functions

We proposed a multiobjective model considering the variability and relationship between economic and environmental aspects (Maximization of Net Present Value (NPV) and ${CO}_{2}$ emissions). Equation (1) shows the objective function of the NPV for which all the cash flows per year must be added. Equation (2) shows the present cash flow values, given that a discount rate for the company's investments must be considered.

(1) $$\mathrm{Max}\;NPV=\sum_{\forall j}{FEA}_{j}$$

(2) $${FEA}_{j}=\frac{{FE}_{j}}{{\left(1+ir\right)}^{j}}\forall j=0,1,\ldots ,J$$

Equation (3) shows the second objective function (minimization of ${CO}_{2}$) for the used transportation by the producers to take the milk to the different collection centers. The calculation of the used motorcycles' engine work for milk transportation is considered (see Appendix A-2) and relates it to the consumption of gasoline per distance traveled. We considered the work performed by the motorcycle and the slope used for the distance traveled by each producer going to the corresponding cluster.

(3) $$\mathrm{Min}\;{CO}_{2}=\left(E1*E2\right)\sum_{\forall i}\sum_{\forall m}\sum_{\forall t}({W1}_{i,m,t}+{W2}_{i,m,t}+{W3}_{i,m,t}+{W4}_{i,m,t})*\left(D_{i,m}*W_{m,i,t}\right)$$

#### 3.2.6 Constraints

The cash flow is calculated considering the fraction of depreciable capital and the company's net annual earnings, as shown by constraint (4).

(4) $${FE}_{j}=\left(1-ti\right)*{NetProfit}_{j}\forall j=1,2,\ldots ,J$$

$${FE}_{j}={InvIn}_{j}\forall j=0$$

Net profit (5) is the result of the difference between gross profit and taxes:

(5) $${NetProfit}_{j}={GrossProfit}_{j}-{Taxes}_{j}\forall j=1,2,\ldots ,J$$

Constraints (6)–(7) show operating and gross profits calculation. Equations (8), (9) show the calculation of miscellaneous and administrative expenses.

(6) $${OperatProfit}_{j}={GrossProfit}_{j}-{GVarios}_{j}-{GAdmon}_{j}\forall j=1,2,\ldots ,J$$

(7) $${GrossProfit}_{j}={VenT}_{j}-{CVentas}_{j}\forall j=0,1,\ldots ,J$$

(8) $${GVarios}_{j}={GVarios}_{j-1}+{VenT}_{j}*PGV-{PGVarios}_{j}\forall j=0,1,\ldots ,J$$

(9) $${GAdmon}_{j}={GAdmon}_{j-1}+{VenT}_{j}*PGA-{PGAdmon}_{j}+{GInteres}_{j}\forall j=0,1,\ldots ,J$$

Equation (10) shows that the initial investment is given by the value of the infrastructure and the installed tanks, the purchase of cattle, machinery, and computer and office equipment. It only occurs during the initial period. Equation (11) shows the value of taxes for each period.

(10) $${InvIn}_{j}=\sum_{\forall m}\sum_{\forall t}\left({vinf}_{t}+{val}_{t}\right)*Z_{m,t}+Vac+Maq+Eco+Eof\forall j=0$$

(11) $${Taxes}_{j}={GrossProfit}_{j}*PIMP\forall j=1,2,\ldots ,J$$

We have considered the sales costs associated with the inventories of concentrate for the animals and fertilizer for pastures, employee salaries, financial obligations and the cost of transportation used to transport the milk to the enabled cluster, the refrigeration costs of stored milk, and depreciation costs (12).

(12) $${CVentas}_{j}={InvFin}_{j-1}+{Inv}_{j}+{Obligac}_{j}-{InventaF}_{j}+{CTransp}_{j}+\sum_{\forall i}\sum_{\forall m}\sum_{\forall t}{CRF}_{t,j}*{Ofer}_{i,j}*W_{m,i,t}+\sum_{\forall m}\sum_{\forall t}\left({CA}_{t,j}+{CIN}_{t,j}+Sue\right)*Z_{m,t}\forall j=1,2,..,J$$

Equations 13–16 show the calculation of some expenses related to the company's economic activity; the details of these calculations are shown in the parameters of appendix A-1.

(13) $${PGVarios}_{j}={GVarios}_{j-1}\forall j=1,2,\ldots ,J$$

(14) $${PGAdmon}_{j}={GAdmon}_{j-1}\forall j=1,2,\ldots ,J$$

(15) $${GInteres}_{j}={Inter}_{j}\forall j=1,2,\ldots ,J$$

(16) $${PImp}_{j}={Taxes}_{j-1}\forall j=1,2,\ldots ,J$$

Equation (17) consider the transportation cost of using motorcycles or on foot

(17) $${CTransp}_{j}=\sum_{\forall m}\sum_{\forall t}{Ofer}_{i,j}*D_{i,j}*W_{m,i,t}*\left[\left(CTra*{Feet}_{i}\right)+\left(TrMot*{Moto}_{i}\right)\right]\forall i=1,2\ldots ,I$$

Constraints (18) allow controlling the location of tanks with smaller capacity with the offer generated by each producer belonging to each cluster. In particular, the offer in each cluster is sought less than the enabled tanks' capacity.

(18) $$\left(\sum_{\forall i}{Ofer}_{i,j}*W_{m,i,t}\right)\leq \left({Capt}_{t}*Z_{m,t}\right)\forall i=1,2\ldots ,I$$

Equation (19) allow the offer for each cluster must be equal to or greater than a minimum percentage of use of the capacity of the tanks.

(19) $$\left(\sum_{\forall i}{Ofer}_{i,j}*W_{m,i,t}\right)\geq PCT*\left({Capt}_{t}*Z_{m,t}\right)\forall i=1,2\ldots ,I$$

Constraints (20) indicate the cluster to which each producer i belongs. Note that a producer can only belong to one cluster.

(20) $$\sum_{\forall m}\sum_{\forall t}W_{m,i,t}=1\forall i=1,2\ldots ,I$$

Constraints (21) indicate that open clusters could have only one tank. Constraints (22) ensure the epsilon constraint approach for objective function (3).

(21) $$\sum_{\forall t}Z_{m,t}\leq 1\forall m=1,2\ldots ,M$$

(22) $${CO}_{2}\leq epsilon$$

### 3.3 Metodología FePIA

The main objective of this research is to design a model for the sustainable evaluation of a raw milk collection system. This methodology can be defined as a series of steps that provide a way to measure the robustness of any system considering its system resources, operational characteristics, and the effects of previously defined disturbance parameters.

FePIA methodology (Features, Perturbation, Impact, and Analysis) to analyze the robustness of a system was proposed by Ref. [34]. The procedure was modified accordingly to the case study. Below is described the adjusted methodology used to analyze the system's capacities in terms of sustainability. We perform the calculation of the system robustness:

i. Definition of the robustness requirements system.
ii. Definition of the system's operating characteristics regarding the system robustness requirements and tolerances.
iii. Determination of the disturbance parameters.
iv. Determination of the effect of the disturbance parameters upon the different operating characteristics.
v. Determination of the effect of disturbance parameters upon the different requirements of disturbance of the system.

The FePIA methodology selects the robustness requirement (Γ), which establishes whether the studied system is robust. Once the robustness requirement is defined, the performance characteristics of the system (Φ) must be determined. These have significant quantitative variations within the maximum and minimum predefined values $\left\langle \beta_{j}^{\min},\beta_{j}^{\max}\right\rangle$, which allows meeting the robustness requirement. Then, it is necessary to determine the disturbance parameters (Π), which affect the robustness requirement and the established performance characteristics and are generally environmental disturbances such as variations in supply, changes in demand, and interruptions in the system.

#### 3.3.1 Perturbation parameters (Π)

Two disturbance parameters were established for the sustainable design of the raw milk collection system: the supply of raw milk and the price established for selling raw milk. These two factors may vary due to internal and external aspects such as the economy's behavior and competition. The first source of uncertainty considered in this study is associated with the variability of milk production by cattle in the study area. The total annual milk supply is calculated in Equation (23). The second source of uncertainty associated with the variation in prices per liter of milk is calculated using (24).

(23) $$\Pi_{1}=\sum_{\forall i}{Ofer}_{i}$$

(24) $$\Pi_{2}=Prec$$

The design of the proposed logistics network operates during several periods (years) where each one has a defined offer by the annual milk production (${Ofer}_{i}$) for each producer. This value depends on the number of cows (Sims, 2021) and sales prices of raw milk per liter (Prec) according to records from the Monthly Bulletin of Farm Milk Prices of the National Department of Statistics through the Price and Supply Information System of the Agricultural Sector ([109]). The supply varies between the maximum (Ofmax) and minimum (Ofmin) values of annual production (liters of milk) from a cow depending on conditions such as climate, diet, technological infrastructure, and the production system. The same occurs with the sales prices of milk, which vary between the maximum (Premax) and the minimum (Premin) sales prices established in the DANE bulletin for the last five years brought to present value. The variability of raw milk supply and the sale price of raw milk have described a set of discrete scenarios obtaining Equations (25), (26). Equations are composed of a coefficient of variation (δ) that varies from 0% to 100% in intervals of 25%, which results in 4 evaluation scenarios (π).

(25) $${Ofer}_{i}=\left[Ofmin+\frac{\delta \left(Ofmax-Ofmin\right)}{100}\right]*{NBOV}_{i}\forall i=1,2\ldots ,I$$

(26) $$Prec=\left[Premin+\frac{\delta \left(Premax-Premin\right)}{100}\right]$$

Once the above steps are completed, the effects of these variations on the performance characteristics of system (Φ) must be analyzed through experimentation and intentionally controlled variation of the disturbance parameters (Π). Finally, the effect of the intentional controlled variation of the disturbance parameters on the established robustness requirement (Γ) should be reviewed.

#### 3.3.2 Robustness requirement (Γ)

*Robustness requirements* are intervals associated with indicators that show whether the logistics system is working correctly. Given that the designed logistics system must be sustainable, different economic, social, and environmental requirements must be considered. The number of open clusters in the system is the first robustness requirement. A system is robust with many clusters or groups due to the direct relationship with transportation time. If more groups are opened, then less time is invested in transportation. Equation (27) defines the first robustness requirement for the number of open clusters. Note that the value of (27) is high, minor transportation cost is required, then the robustness requirement is satisfied.

(27) $$\Gamma_{1}=\sum_{\forall m}\sum_{\forall t}Z_{m,t}$$

The second robustness requirement considers the value of the profits obtained by the producers. The system is considered robust if, despite the changes presented, the producers maintain their established minimum earnings. Equation (28) shows the producers' minimum profit in the contemplated time horizon (calculations supported in Appendix A-1). Therefore, if the value of (28) is high, the robustness requirement is satisfied because (24) indicates the minimum profit producers obtain.

(28) $$\Gamma_{2}=ValorMinin=\sum_{\forall J}{ProdOperatPro}_{ij}\forall i=1,2,3\ldots I$$

It is necessary to add constraints (29) and (30) to determine the minimum value of (28). These constraints guarantee that the calculated value corresponds to the minimum earnings of all producers.

(29) ValorMin=Variab

(30) $$\sum_{\forall j}{ProdOperatPro}_{ij}\text{:}-Variab>0\forall i=1,2\ldots ,I$$

Another robustness requirement for the system is the system's CO_2_ emissions from transporting raw milk, supported by Equation (18). The system is considered robust for low values of CO_2_ emissions. Equation (31) calculates the total average distance traveled by each producer j if it must travel to the cooling tank by any transportation mode. Equation (32) calculates the average distance traveled by the producer by using a motorcycle. Finally, Equation (33) calculates the producer's average traveled distance on foot. Thus, the designed system is considered robust for shorter distance values.

(31) $$\Gamma_{3}=DistPTotal=\frac{\sum_{i,m,t}D_{i,m}*W_{m,i,t}}{\sum_{i,m,t}W_{m,i,t}}\forall i=1,2\ldots ,I\forall m1,2\ldots ,M\forall t=1,2\ldots ,T$$

(32) $$\Gamma_{4}={DistPromMoto=Moto}_{i}*\frac{\sum_{i,m,t}D_{i,m}*W_{m,i,t}}{\sum_{i,m,t}W_{m,i,t}}\forall i=1,2\ldots ,I$$

(33) $$\Gamma_{5}={DistPromFeet=Feet}_{i}*\frac{\sum_{i,m,t}D_{i,m}*W_{m,i,t}}{\sum_{i,m,t}W_{m,i,t}}\forall i=1,2\ldots ,I$$

#### 3.3.3 System performance characteristics (Φ)

The performance characteristics must have a limited variation to meet the robustness requirements described above. Three performance characteristics were considered for the model described above: 1. Installed Milk Collection Capacity (lts/year), 2. Cost of energy for cooling tanks (COP/year), and 3. Utilization of storage capacity of cooling tanks. Equation (34) calculates the total capacity of tanks for each configuration according to the enabled open clusters.

(34) $$\varphi_{1}=CapTot=\sum_{\forall m}\sum_{\forall t}{Capt}_{m,t,}*Z_{m,t}$$

Equation (35) calculates the utilization percentage of each tank for each configuration, taking into account the clusters enabled at the same time, the capacity of the type of tank, and the annual supply of each producer j.

(35) $$\varphi_{2}={Util\_Tanq}_{m,t,j}=100*\frac{\sum_{\forall i}{Ofer}_{i,j}*W_{m,i,t}}{{Capt}_{m,t,}*Z_{m,t}}$$

Equation (36) shows the calculation of the energy cost associated with the refrigeration of a liter of milk according to the type of enabled tank.

(36) $$\varphi_{3}=C\_Energy=\sum_{\forall i}\sum_{\forall j}\sum_{\forall m}{Ofer}_{i,j}*W_{m,i,t}*{CRF}_{t,j}$$

We defined a final stage considering a tactical plan for using the installed tanks for the best-established configuration by the robustness-NPV and robustness-CO_2_ relationship. In addition, the methods to use the tanks for the four scenarios concerning the different values of π are established. Compared to the evaluation stage of the impact of $\Pi_{p}$ on $\Gamma_{s}$ and $\varphi_{f}$, the installed capacities of the tanks are fixed. The following modifications must be made to the proposed model: i) it is necessary to create a binary parameter called $a_{m}$ that indicates the construction or not of a tank in a specific location of one of the producers: 1 if a tank is located in producer i, which belongs to the cluster m, and 0 otherwise, and ii) a new associated constraint with the location control of the enabled clusters must be created. Therefore, a binary variable called $B_{m}$ is created, which indicates whether the cluster is activated for the current design. With $B_{m}$, constraint (21) must be replaced by (37):

(37) $$\sum_{t}Z_{m,t}=a_{m}*\;B_{m}$$

When creating the new cluster activation parameter according to the selected configuration, it is necessary to change the calculation of the milk sales costs. Unlike Equation (11), the costs associated with tank and infrastructure depreciation were always considered (38) and did not depend on whether the tank was used.

(38) $${CVentas}_{j}={InvFin}_{j-1}+{Inv}_{j}+{Obligac}_{j}-{InventaF}_{j}+{CTransp}_{j}+\sum_{\forall i}\sum_{\forall m}\sum_{\forall t}{CRF}_{t,j}*{Ofer}_{i,j}*W_{m,i,t}+\sum_{\forall m}\sum_{\forall m}Sue*Z_{m,t}+\sum_{\forall t}\left({CA}_{t,j}+{CIN}_{t,j}\right)\forall j=1,2,\ldots ,J$$

Constraints (19) are deleted because the use of minimum tank capacity is no longer tactically needed. During periods of low milk production, certain tanks would not be used or would only be filled to a small percentage of their actual capacity.

**Fig. 1:**
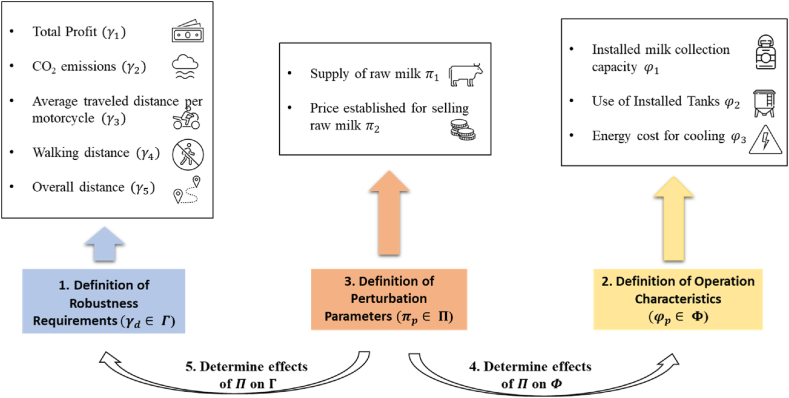
FePIA methodology.

According to the previous methodology, we present a general scheme for measuring raw milk collection system robustness (Fig. 1):

## 4 Computational experiments

**Fig. 2:**
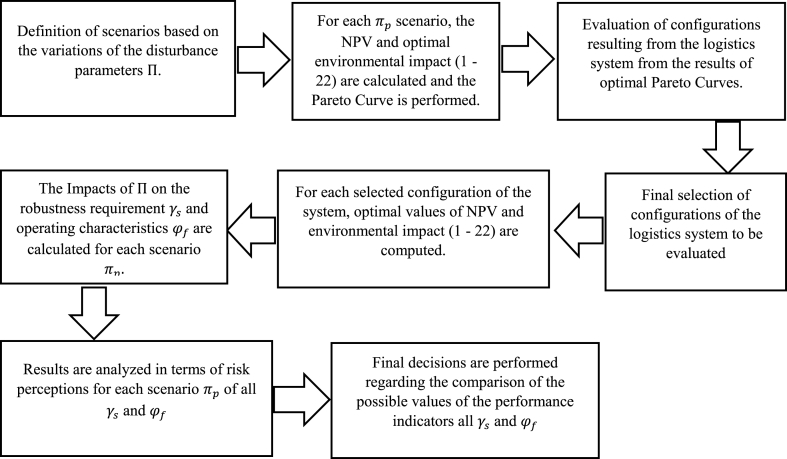
Flowchart of the technical procedure for robustness analysis.

The Flowchart (Fig. 2) describes a technical procedure for the robustness analysis of the raw milk collection system.

Step 1 The disturbance parameters (Π) are varied in this stage in a controlled manner using equations (25), (26) by generating different evaluation scenarios to identify the robustness requirements Γ and the performance characteristics φ.

**Step 2.** Based on the model described above, the performance characteristics were considered using equations 27–36. The robustness requirement is an interval associated with one or more indicators, revealing whether the logistics system works correctly. The performance characteristics needed limited variation to the described robustness requirements to be satisfied. The mathematical model described above is used to determine from each variation of Π the Pareto curves and configurations of the logistic system.

Step 3 The results of step 2 specified various configurations of the logistic system concerning the variations of Π. Each configuration is described to determine similarities to group them.

Step 4 Once the different configurations of the logistics system have been gathered, a single configuration is determined for each group. For example, the configurations gathered in a group vary by the tank layout in a producer for a distance of fewer than 250 m. A single position of that tank is selected.

Step 5 Constraints (37–38) have been added to the optimization model forcing it to adopt each configuration selected from the previous step. Subsequently, for each configuration, the disturbance parameters (Π) are varied again according to the values considered in Step 1. Therefore, the following modifications have been performed to the proposed model:

1) Introduced a new known parameter $\left(a_{m}\right)$ that indicates the construction or not of a tank for a specific location of one of the producers: 1 if a tank is located in producer i, which belongs to cluster m, and 0 otherwise, and ii) a new constraint associated with the location must be created controlling the enabled clusters. Therefore, a binary variable called $B_{m}$ is created, which indicates whether the cluster is enabled for the current layout. With $B_{m}$, constraint (21) must be replaced by (37).
2) Constraints (19) are removed because the minimum tank capacity is no longer tactically necessary. During periods of low milk production, certain tanks would not be used or would only be filled to a small percentage of their actual capacity.
3) Constraints (12) are updated by Constraints (38) since ${CA}_{tj}$ and ${CIN}_{tj}$ do not depend on the binary variable $Z_{mt}$.

Step 6 The relationships of the different parameters were graphically analyzed once Γ and Φ were determined separately for each configuration according to the controlled variation of the scenarios resulting from each variation of Π.

**Steps 7** and **8** indicate the analysis and decision-making resulting from applying the FePIA procedure.

### 4.1 Case of study

The efficiency of the proposed methodology was tested for designing a logistics system for collecting raw milk in a Colombian company located in Bogotá. This company distributes raw milk to different processing plants and requires a study to request economic resources in departmental development plans. Currently, the company is evaluating the structure of the collection logistics network that maximizes the NPV of the company's economic resources and considers other sustainable aspects. The company has evaluated the possibility of installing different cooling tanks.

The system's robustness requirements were first determined as the company's total income from the sale of milk, the number of CO_2_ emissions from using motorcycles in the milk transport, and the distances traveled by the producers to transport the raw milk to the refrigeration tank. The performance characteristics of the raw milk collection system that must be maintained in the robust logistics network design are the storage capacity of the refrigeration tanks to be installed and the energy cost of the tanks. Finally, the amount of milk offered by each of the company's producers and the milk sales prices have been established as system disturbance parameters that affect the performance measures and the characteristics that make the system robust.

### 4.2 Analysis of results

The robust design of the raw milk collection logistics system is based on the proposed mathematical model. The problem was coded in version 22.5 of the GAMS and CPLEX solver software on a computer with an AMD A9 processor running 3.6 GHz with 8 MB of RAM.

Initially, maximum and minimum values are calculated using the obtained solutions according to the objective functions. The objective functions are standardized to respond to the evaluation stage of the impact of $\Pi_{p}$, $\Gamma_{s}$, and $\Phi_{f}$ and equivalent values between δ and π. These values remained constant for the rest of the experiment. Given the number of producers and their size of cattle, the minimum volume of milk that could have been produced in a year was 1,800 lt/cow-year, while the maximum volume could reach 3,600 Lt/cow-year. It is essential to highlight that of the eight scenarios considered, only four were used since the sale price of raw milk did not influence the results of the $\Gamma_{s}$ values. Only the maximum milk values were worked on since the study's objective is that the companies maintain this price due to compliance with the cold chain maintenance policies. Table 2 shows the best and worst objective function values obtained from the individual optimization method.

**Table 2:** Best and worst objective function values obtained from the individual optimization method.

	Scenario $\pi_{1}$	Scenario $\pi_{2}$	Scenario $\pi_{3}$	Scenario $\pi_{4}$
$Offer\left(\frac{lt}{cow-year}\right)$ $Price\;\left(\frac{Cop}{lt}\right)$ ValueOF	1800	2400	3000	3600
	1300	1300	1300	1300
**Max Kg CO**_**2**_	79,75	80,34	57,01	62,86
**Min Kg CO**_**2**_	31,42	30,22	23,76	21,15
**Max NPV COP**	450,212 × 10^6^	546,630 × 10^6^	644,537 × 106	742,624 × 106
**Min NPV COP**	444,443 × 10^6^	540,549 × 10^6^	636,730 × 106	732,615 × 106

Preliminary results on the evaluation of the impact of $\Pi_{p}$ on $\Gamma_{s}$ indicate that as the value of milk offered by producers increases during the planning period, it becomes necessary to increase the number of installed tanks. Therefore, there is an increment of the $\Gamma_{1}$ values. This situation is similar to the values of minimum profit for associated producers $\Gamma_{2}$ and the total traveled distance of milk transportation $\Gamma_{3}$. However, the motorcycle transportation distance $\Gamma_{4}$ decreases because the installed tanks are located on producers' farms with this mode of transportation. Likewise, the distance of milk transport on foot $\Gamma_{5}$ is increased, thus causing a problem in people's health.

The evaluation of $\Pi_{p}$ on $\Phi_{f}$ indicates that the total installed capacity of the tanks $\varphi_{1}$ increases, and the energy cost $\varphi_{2}$ increases due to the installation of additional tanks. However, the use of tanks $\varphi_{3}$ decreases because tank sizes are smaller and are sometimes only used at their minimum stated capacity (70%). Subsequently, the epsilon constraint (ε) method is used to solve the multiobjective model, which optimizes the economic objective by restricting the environmental impact objective within a specified range by the user ([110]). In the ε-constraint method, the highest priority function was assumed to be the primary objective (Net Present Value), and the other objective function was treated as a constraint. The constraint limits are given by an epsilon vector varying between the maximum and minimum values of each scenario. The assumptions of these results are the same as the proposed model to evaluate the logistics system.

**Fig. 3:**
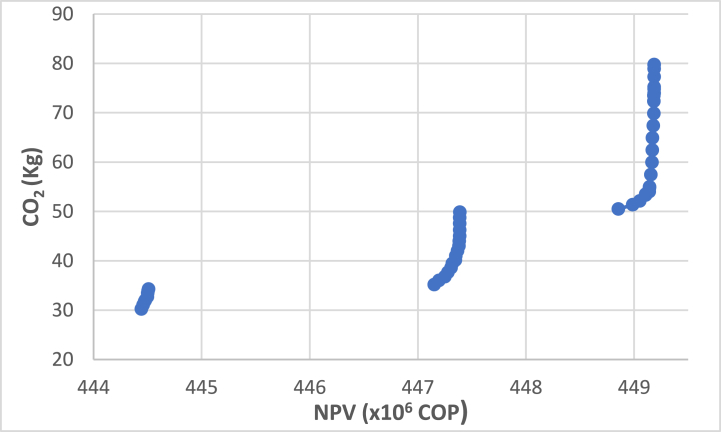
Pareto optimal curve for scenario $\pi_{1}$.

**Fig. 4:**
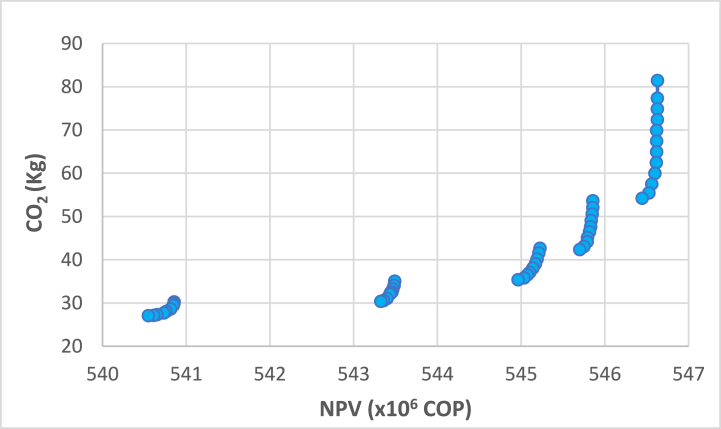
Pareto optimal curve for scenario $\pi_{2}$.

**Fig. 5:**
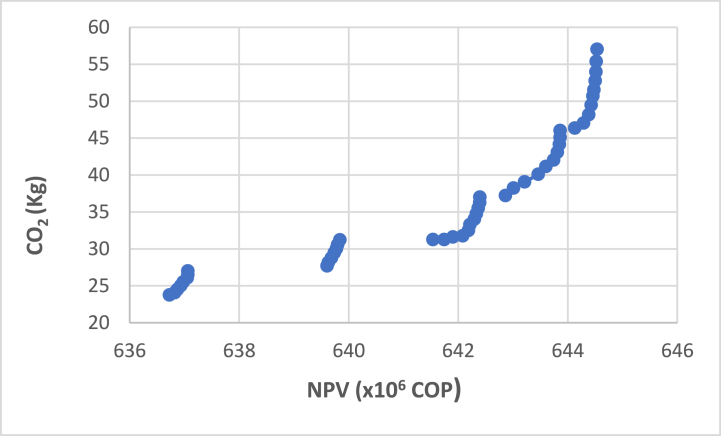
Pareto optimal curve for scenario $\pi_{3}$.

**Fig. 6:**
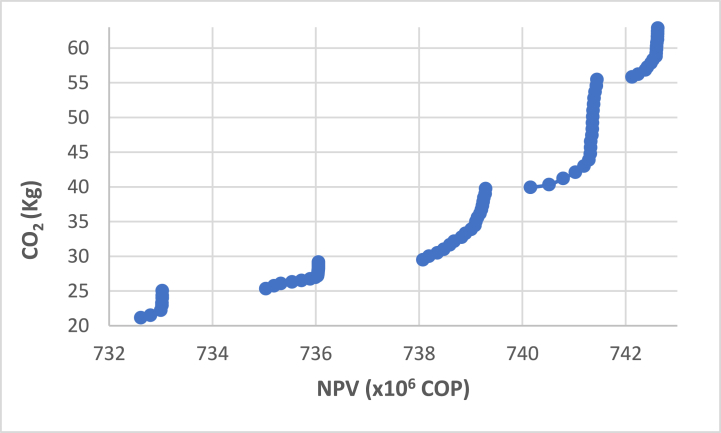
Pareto optimal curve for scenario $\pi_{4}$.

The Pareto optimal curves for the different π scenarios are shown in Fig. 3, Fig. 4, Fig. 5, Fig. 6. Various values of NPV and GHG emissions vary in the figures. Pareto optimal curves indicate NPV values that cannot be obtained. For these cases, reducing the amounts of emitted CO_2_, there are decreases in NPV values due to changes in tank configurations and values represented in the initial investments for the milk collection logistics system.

**Fig. 7:**
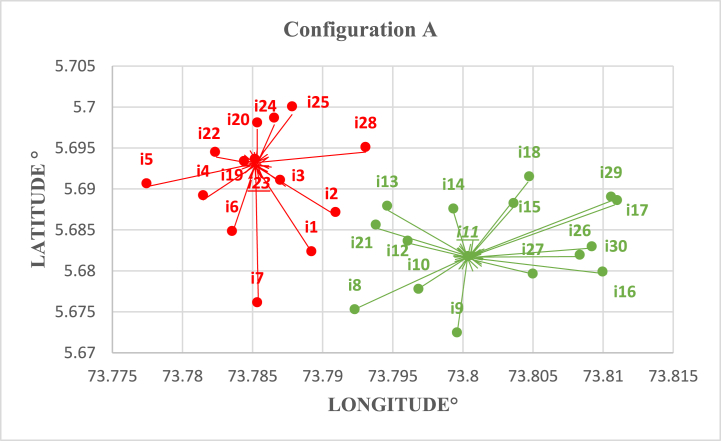
Configuration A for the supply chain system.

**Fig. 8:**
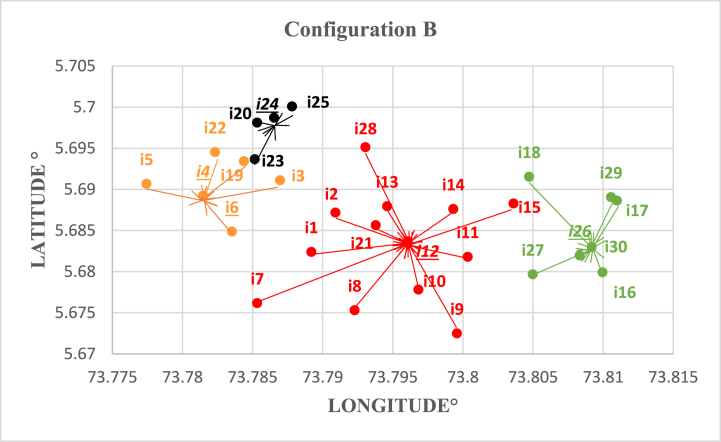
Configuration A for the supply chain system.

**Fig. 9:**
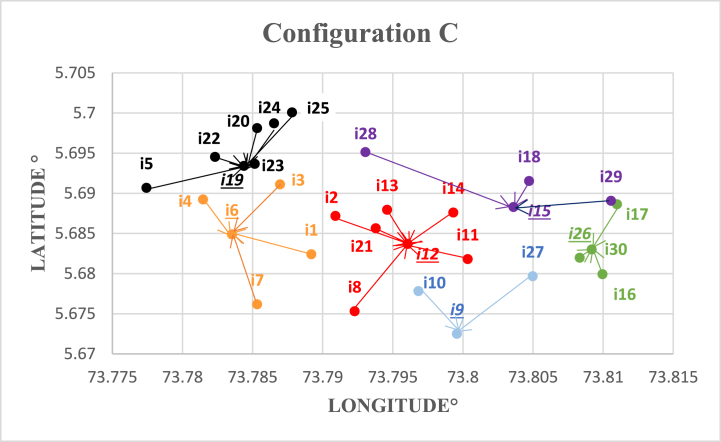
Configuration C for the supply chain system.

**Fig. 10:**
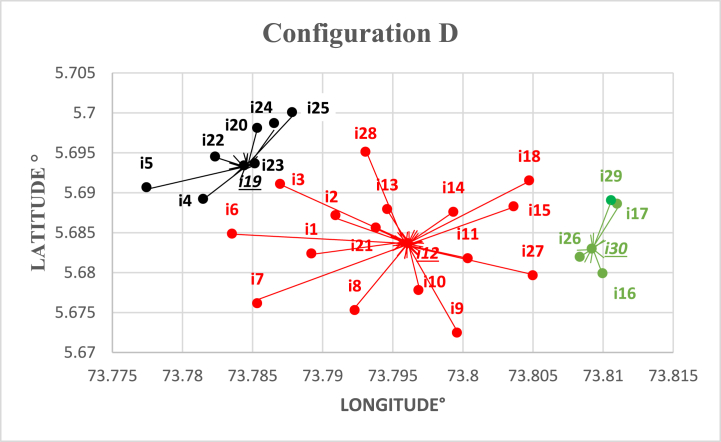
Configuration D for the supply chain system.

**Fig. 11:**
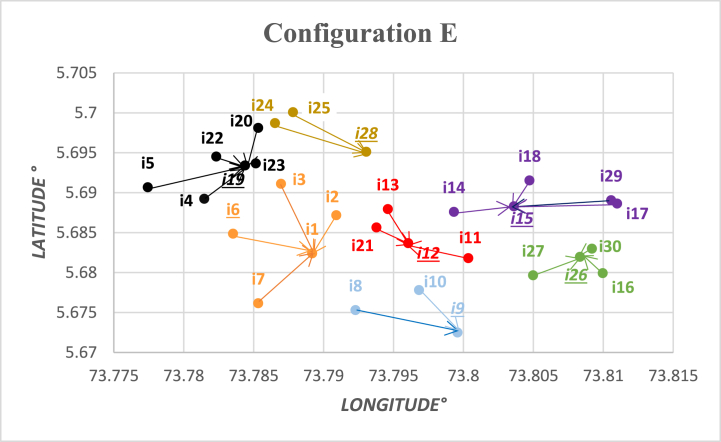
Configuration E for the supply chain system.

After obtaining the Pareto chart for each scenario, the configurations obtained by similarity were grouped, obtaining 28 configurations. Most of the resulting configurations vary only in opening another cluster with a lower capacity tank, which can be considered the same configuration for the strategic plan. Six configurations of the 28 possible configurations were selected. The final selection of configurations was performed based on the maximum capacity used and the proximity of the tanks that are not used with low demand. Fig. 6, Fig. 7, Fig. 8, Fig. 9, Fig. 10, Fig. 11 show the six configurations adopted by the system after the computational tests. The tank location of a group is indicated by a point where a blue dot marks the producer. These configurations are labeled A–F in Table 4 and their relationship to the parameter π. These configurations are later evaluated with the FePIA methodology to determine their level of robustness.

### 4.3 Scenario analysis and the effect of disturbance parameters on operating characteristics

For the analysis of scenarios for evaluating disturbance parameters in operation characteristics and robustness requirements, steps 5-8, described in Fig. 2, are used. For the evaluation of possible scenarios, the following assumptions have been taken into account:

• The chosen configurations are evaluated for each scenario.
• The number of raw milk cargo vehicles cannot vary for each evaluation.
• The number of milk-producing cows is maintained for each producer.

Once the variations of the disturbance parameters were used to determine the different scenarios, the total installed capacity of the tanks, refrigeration cost, and percentage of use of the tanks' capacity were analyzed. These characteristics were selected as system performance features.

#### 4.3.1 Configuration A

Two clusters are enabled for producers 23 and 27, and tank type 4 must be installed. These tanks have an installed capacity per year of 1,152,000 L/per year, and their percentage of use is 97.33%. Its representation corresponds to Fig. 7.

#### 4.3.2 Configuration B

Four clusters are enabled for producers 4, 12, 24, and 26. Tank type 1 must be installed in producers 4, 24, and 26, while tank type 4 must be installed in producer 12. The total installed capacity is 1,137,600 L/year, and its use percentage is 98.12%. Its representation corresponds to Fig. 8.

#### 4.3.3 Configuration C

This configuration has 6 clusters enabled for producers 6, 9, 12, 18, 19, and 26. Only tank type 4 is installed for all producers, whose installed capacity per year is 3,456,000 L/per year, and their use percentage is 96.45%. Its representation corresponds to Fig. 9.

#### 4.3.4 Configuration D

Configuration D (Fig. 10) considers three enabled clusters for producers 12, 19, and 30. Tank type 3 is enabled for Cluster 12. Tank type 2 is installed for Cluster 19. Finally, tank type 1 is installed for producer 30. The installed capacity per year is 853,200 L/year, and its use percentage is 98.13%.

#### 4.3.5 Configuration E

Seven clusters are enabled for producers 1, 9, 12, 18, 19, 26, and 28. Tank type 1 is enabled for all cases considering an installed capacity per year of 1,310,400 L, and its percentage of use is 97.58%. Its representation corresponds to Fig. 11.

#### 4.3.6 Configuration F

**Fig. 12:**
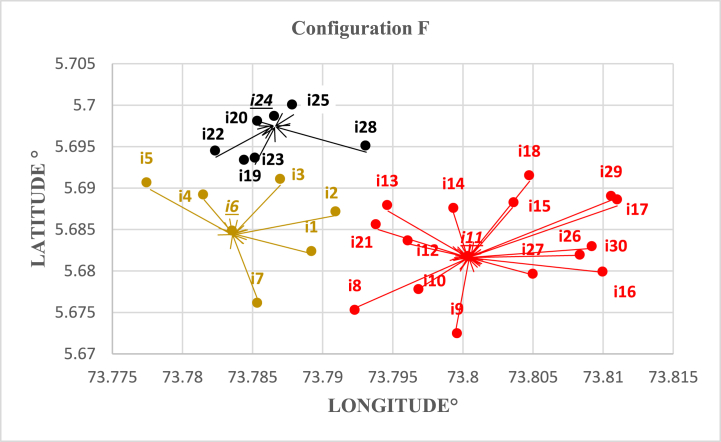
Configuration F for the supply chain system.

Finally, for configuration F (Fig. 12), three clusters are enabled for producers 6, 24, and 11. Tanks types 1, 2, and 5 are located, and the installed capacity per year is 1,141,200 L/year, and its percentage of use is 98.83%.

### 4.4 Robustness Requirements Analysis (Γ)

The proposed robustness requirements have been analyzed to evaluate the system's performance through the proposed model. Economic, social, and environmental aspects have been considered. The different levels of uncertainty associated with the disturbance parameters are addressed through four scenarios, from the most pessimistic (lower annual milk supply with a minimum sale price) to the most optimistic (higher milk supply with a higher sale price). Table 3 lists the obtained results from the scenarios. Note that in the most optimistic scenario, better benefits are obtained. However, for this scenario, robustness requirements associated with social aspects (the effort to walk through canteens) or environmental aspects (use of motorcycles or CO_2_ emissions) also increase. Each is analyzed for each scenario proposed to determine the most robust configuration. The results show that the system's performance varies depending on the analyzed robustness requirement (Γ). No configuration obtains the best performance in each requirement Γ for each scenario π. In this sense, it is necessary to review the information summarized in Table 3 to determine which configuration presents the best performance for the different values of Γ.

**Table 3:** Obtained results for robustness requirements.

Configuration	Total Profit (COPx10^6^/year-productor)				CO_2_ emissions (Kg CO_2_/year)				Overall Distance (Km/year)				Motorcycle distance (Km/year)				Walking distance (Km/year)			
	π_1_	π_2_	π_3_	π_4_	π_1_	π_2_	π_3_	π_4_	π_1_	π_2_	π_3_	π_4_	π_1_	π_2_	π_3_	π_4_	π_1_	π_2_	π_3_	π_4_
Conf. A	19.370	22.906	26.508	30.055	76.055	80.348	48.760	24.062	11413.59	11493.61	9802.30	10516.14	14357.18	14427.21	9204.60	4542.27	8470	8560	10400	16490
Conf. B	17.453	21.943	25.225	27.056	70.408	70.564	48.880	27.144	10978.39	10993.39	9277.35	9386.31	13546.77	13576.79	9404.70	5222.61	8410	8410	9150	13550
Conf. C	18.219	22.429	25.980	29.960	68.563	69.986	45.849	22.608	11158.25	11433.32	9707.18	9421.07	13016.51	13286.64	8704.35	4292.15	9300	9580	10710	14550
Conf. D	19.008	22.669	26.388	29.052	71.553	71.656	46.737	23.213	11048.46	11173.47	9377.26	9021.12	13846.92	13866.93	9044.52	4492.25	8250	8480	9710	13550
Conf. E	20.452	23.829	28.157	31.855	62.825	66.433	40.631	21.155	10423.14	10573.32	8412.03	8436.08	12546.27	13266.63	8114.06	4322.16	8300	7880	8710	12550
Conf. F	19.920	23.519	27.756	30.687	74.712	75.296	51.135	31.435	11123.52	11103.55	9287.41	9261.48	14077.04	14187.09	9634.82	5922.96	8170	8020	8940	12600

**Table 4:** Results for system performance characteristics (Φ).

	Cap. Utilization Ratio (%)					
Esc.	Conf. A	Conf. B	Conf. C	Conf. D	Conf. E	Conf. F
π_1_	56.88	54.07	56.92	56.33	48.79	56.33
π_2_	71.10	67.59	71.15	70.42	60.99	70.42
π_3_	85.32	81.11	85.38	84.50	73.18	84.50
π_4_	99.55	94.63	99.61	98.59	85.38	98.59
Installed Capacity (L/Year)	1232000	1296000	1231200	1244000	1436400	1244000

#### 4.4.1 Total Profit $\left(\Gamma_{1}\right)$

The analysis of this robustness requirement shows that for the different scenarios analyzed, the total profit per product in one year varies from COP 17,453,166 (scenario $\pi_{4}$) for configuration B to COP 31,854,717 (scenario $\pi_{1}$) for configuration E.

**Graph 1:**
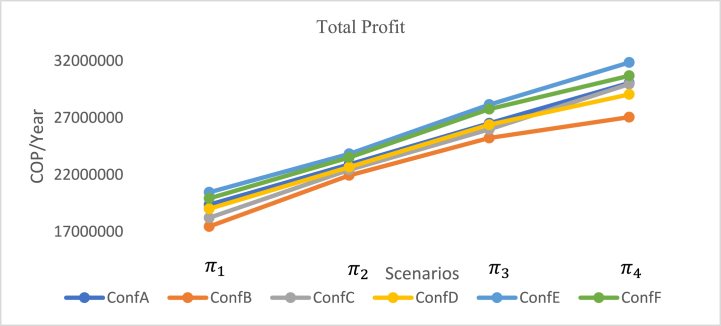
Obtained results for $\Gamma_{1}$ 4.4.2. CO2 emissions $\left(\Gamma_{2}\right)$.

Fig. 1 shows that configuration E presents the highest annual profits for raw milk producers. In this sense, this configuration is optimal for maximizing its income. If scenario $\pi_{1}$ occurs, then profits will drop per producer to COP 27.055.515/year, representing a decrease of 15.07% of the maximum obtained for scenario $\pi_{4}$ Graph 1.

The behavior of CO_2_ emissions is directly related to using motorcycles as a transportation mode, the transported weight, and the slope of the road. If the milk supply is high, then more trips must be performed, the engine's work is increased, and more emissions are emitted into the air. Configuration E positions the cooling tanks at a lower height above sea level, allowing the transported weight to be performed downward. Therefore, it provides better results for all scenarios. Graph 2 shows the possible configuration behavior for all scenarios. Configuration A presents the worst results for scenarios $\pi_{1}$ and $\pi_{2}$.

In scenario $\pi_{2}$, the configurations present the maximum results, reaching a maximum of 80.35 KgCO_2_/year for configuration A. In contrast, configuration E for scenario $\pi_{4}$ generates up to 21.16 KgCO_2_/year, achieving a reduction of 73.67% compared to the worst result. The logistics system resulting from the analysis of the different scenarios Π shows that a reduction of CO_2_ emissions must employ a majority of the use of human force when there is a greater amount of milk produced.

#### 4.4.3 Average traveled distance per motorcycle $\left(\Gamma_{3}\right)$

**Graph 3:**
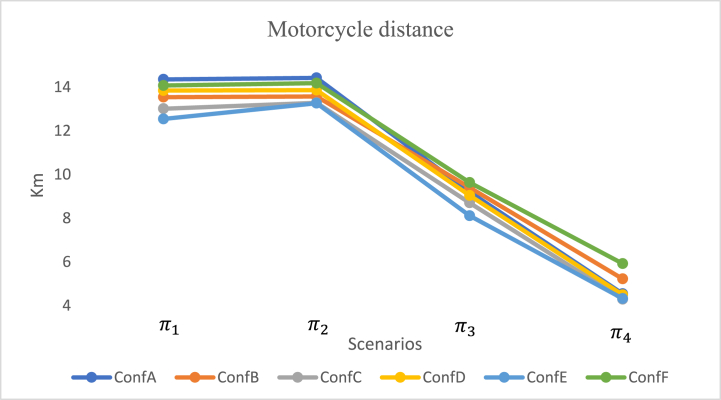
Obtained results for $\Gamma_{3}$ 4.4.4. Walking distance $\left(\Gamma_{4}\right)$.

The behavior of this robustness requirement is related to the number of emissions. The behaviors of Graph 3 are similar to those of Graph 2. Configuration E presents the most significant variation, 5.43%, compared to scenario $\pi_{1}$. Despite this difference, this configuration continues to be the best, slightly higher by 0.15% with configuration C. For scenario $\pi_{4}$, the minor average distance of the used motorcycle is achieved by configuration C with 32 km traveled per day, which is slightly better than configuration E by 0.69% for the total kilometers traveled per day. When analyzing the results of this parameter, the company should adopt configuration E since for scenarios $\pi_{1},\pi_{2}$, and $\pi_{3}$ it presents shorter distance traveled by the motorcycle used in the transport of raw milk, and in the remaining scenario, its increase is not significant concerning the best result of configuration C.

**Graph 4:**
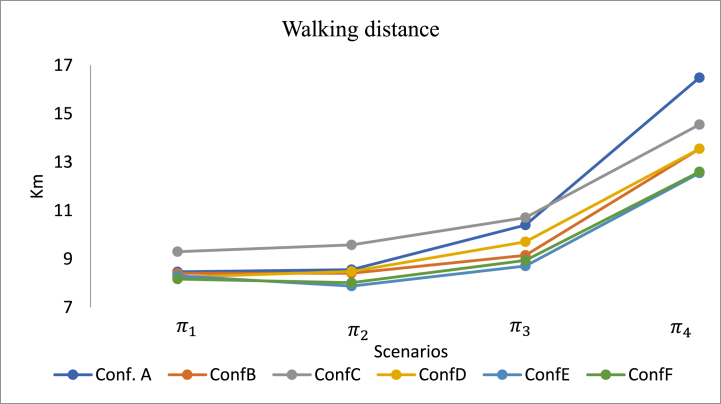
Obtained results for $\Gamma_{4}$.

All configurations were analyzed to increase the distance traveled to transport raw milk canteens. As the weight of canteens full of raw milk increases, the logistics system does not allow motorcycles to be used to reduce CO_2_ emissions. As the human effort for transporting raw milk was not considered by the optimization model used to determine the most robust configuration (only as an indicator of the result), the distance traveled increases as the scenarios are analyzed to increase the amount of produced milk. Configuration F has the shortest distance traveled by partners in manual raw milk transport (or transport on foot) in scenario $\pi_{1}$ at 8.17 km/day. In the remaining scenarios, configuration E has the lowest records of manual transportation of raw milk, but with slight variation from configuration F, on average, 0.017% of the daily mileage covered by configuration E. The results can be seen in Graph 4.

#### 4.4.5 Overall distance $\left(\Gamma_{5}\right)$

**Graph 5:**
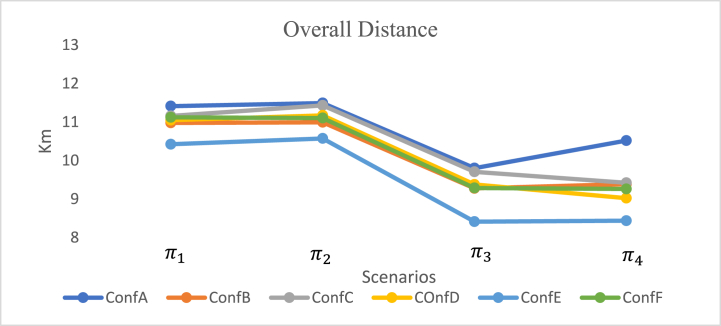
Obtained results for $\Gamma_{5}$ 4.5. Analysis of system performance characteristics (Φ).

The general average of distances traveled in the transport of milk is lower in configuration E. The results can be observed in Graph 5. The average distance traveled in transporting canteens with raw milk either by motorcycle or manually decreases to scenario three, where the milk supply is three-quarters of the total. When the milk supply is at a maximum, the general distances increase due to increased product transportation on foot. The locations of the cooling tanks (8) are closer to the producers who use motorcycles due to the weight of the canteens with raw milk of this transportation mode. This aspect also favors the routes made in manual transport (on foot). Despite being greater due to the disuse of the motorized mode of transport, the routes are not of considerable distances, on average 515 m. This same effect is faced by producers who do not have a mode of motorized transport.

Three performance characteristics have been considered for the proposed model, each of which is a global performance indicator for the company. The milk collection and refrigeration tank's installed capacity ($\varphi_{1}$) was selected because, in any scenario, the tanks must receive all the milk offered. The company will lose money in non-chilled milk if the capacity is reduced. Since the tanks represent an investment for the company, minimum use must be guaranteed in any scenario ($\varphi_{2}$). Energy costs have a direct impact on the profitability of the company ($\varphi_{3})$. Proper use of the tanks and associated costs will impact the money flow in the analyzed periods.

#### 4.5.1 Installed milk collection capacity $\varphi_{1}$

In order to determine the possible configuration adopted by the company in the case study regarding the Installed milk collection capacity $\varphi_{1}$, it has been chosen that all the selected tanks have sufficient capacity for milk storage. According to the case information, the maximum milk supply in scenario $\pi_{4}$ is 1,226,400 L/year. Table 4 shows all possible configurations that can store the maximum milk supply. For this reason, all configurations meet this performance characteristic of the raw milk collection system.

#### 4.5.2 Use of installed tanks $\varphi_{2}$

The use of tanks is greater in scenario π_4_ due to the amounts of milk stored in the scenario with the highest milk supply. For configuration E, seven clusters are created with tanks with a capacity of 205,200 l/year each. The installation of this number of tanks favors the robustness requirements (Γ) but not the performance characteristics. As seen in Table 4, for all scenarios (Π), the tanks' use percentages are lower in configuration E. With requirements of at least 50% use of the installed tanks, this configuration would not comply in the scenario with less supply of milk ($\pi_{1}$).

Six tanks with a minimum capacity of 205,200 l/year are located in $i_{6},i_{9}\text{,}$ $i_{12}\text{,}$ $i_{18}\text{,}$ $i_{19}$ and $i_{26}$ with a used capacity of 99.61% for scenario $\pi_{4}\text{.}$. Configuration A, with two installed tanks with a capacity of 61,600 l/year in producers $i_{23}$ and $i_{27}$, respectively, approaches these capacity use values of configuration C with only 0.01% in scenario $\pi_{4}$ and 0.04% in scenario $\pi_{1}$ Under this analysis, all configurations are under the performance characteristic of keeping the raw milk refrigeration tanks in use for more than 50% of the time requested by the company, except for configuration E in scenario $\pi_{1}$.

#### 4.5.3 Energy cost for cooling $\varphi_{3}$

The energy cost for using the different tanks installed in each of the possible configurations in the scenarios analyzed can be seen in Table 4. The lowest costs are found in scenario $\pi_{1}$, which presents the case company's lowest supply of crude milk. In particular, configuration A has the lowest costs since it is a configuration that only uses two tanks. The energy costs for cooling for this configuration vary between COP/year 20778720 and COP/year 36362760. In contrast, configuration E has the highest cooling costs, varying between COP/year 35525620 and COP/year 62169834, representing an increase of 70.97% concerning configuration A. These high costs can be explained by the number of tanks installed for this configuration, 7, against the 2 of configuration A. Configurations C and F, which in an analysis of the use of installed tanks $(\varphi_{2}$) have the same percentage, vary in their cost due to the different use of those tanks. Configuration F uses more of the 720,000 l/year capacity tank, 9.94% more than configuration C, which has a higher cooling cost than the 262,000 l/year tanks.

**Graph 6:**
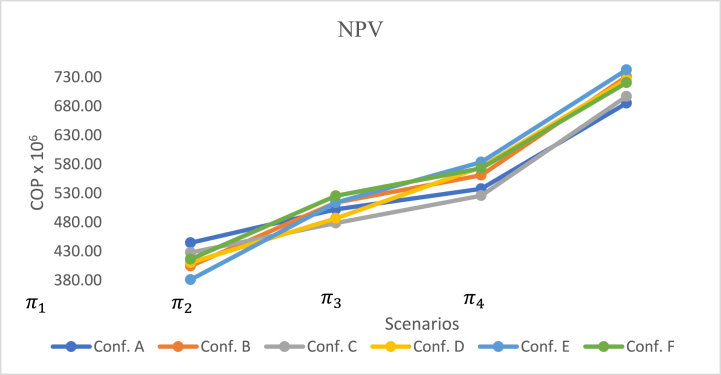
Results for NPV.

The results of the analysis of the performance characteristics of system (Φ) are inconclusive since it is contrary to the result of the robustness requirements (Φ): configuration E has the worst results in the selected performance characteristics. This situation occurs because as a configuration that reduces CO_2_ emissions, it allows the location of 7 cooling tanks that reduce the use of automotive equipment in transporting canteens with raw milk. To make a better decision on which configuration to choose, since this is a strategic decision, it is desired to have a more comprehensive performance characteristic. For this purpose, the first objective function (NPV) is selected since it has all of the different cash flows in a more extended review period than one year. The results for all configurations in all scenarios analyzed (Π) are shown in Graph 6.

As the supply of raw milk increases, the NVP of configuration E improves. This situation is due to the absorption of costs to use resources such as transport modes for milk canteens, use of the infrastructure with a lower depreciation cost, and lower payment of interest on loans for the purchase of infrastructure. In the scenarios $\pi_{1}$ and $\pi_{4}$, the NVP of configuration E is the highest for all options, reaching COP 721524337.54, which is 7.82% higher than the value of configuration A.

### 4.6 Discussion

This work proposed a novel efficient approach to the robust design of sustainable logistics systems for collecting and processing raw milk. The obtained results show the efficiency of making decisions related to strategic decisions, such as the location of facilities (cooling tanks) and assignment of producers to each facility, and tactical decisions, such as the flow of products between nodes (distribution flows). Besides, the paper covers a significant gap related to the analysis of several objectives considering a multiobjective scheme for a sustainable logistics system for collecting and processing raw milk based on environmental and economic aspects. Indeed, the performance of the sustainable logistics system should be based on financial, environmental, and social aspects generating a realistic analysis to support decision-making.

Note that considering the vehicles' load and the road's slope confirm the improvement of the social aspects related to human effort. These aspects highlight a broader social and environmental point of view rather than just considering the economic issues of primary raw milk production. In addition, the results show the collaborative framework's efficiency for decision-making, improving the Sustainable Development Goals (SDG). The need to reduce carbon emissions in agricultural systems has increasingly shown the logistics system's configuration in the spotlight as an effective measure for this problem. In this way, the location of raw milk collection tanks has attracted attention to the sustainable management of these systems. In addition, this location is expected to address the management problem for the long term, as it is a robust configuration. However, the relevant literature shows that researchers have focused more on the robust optimization problem than on integrating robustness metrics in configuring sustainable logistics systems. Additionally, agro-industrial systems that sell products in their first mile have yet to calculate carbon emissions as a corporate strategy.

There must be more than this approach to guarantee carbon reduction throughout the dairy supply chain, as carbon reduction policies must be directly involved with methodology development. Since locating cooling tanks for milk storage is not easy and there is a need to involve additional components in the logistics network, such as the distances traveled by producers, the design faces a more severe uncertainty and consequently a higher cost and increased risk of variation of the supply. Therefore, it is necessary to provide a more comprehensive approach to logistics network design, considering the uncertainty of raw milk supply as a determining factor in a robust way. The interaction between the producers' offer, different used modes of transportation, the capacity of the raw milk collection tanks, and performed investment in its acquisition and maintenance versus the economic risk and generated emissions should be examined. For this logistics system, both dimensions affect the configuration and the network's economic, social, and environmental performance.

Despite the importance of the subject, there needs to be more exhaustive research on first-mile agro-industrial logistics systems integrating all studied characteristics for an appropriate methodology. This paper addresses this research gap by developing a bi-objective MILP model and its solution approach using the FePIA procedure as a robustness metric. The results show that the design of the logistics network, when changing the milk supply, strongly affects the configuration of the network. This effect can be seen in open clusters using different transport modes and storage capacities. The impact on the network configuration directly affects the robustness requirements (Γ) and the performance characteristics of the system (Φ). The results of the analysis of scenarios determined by the variations of $\Pi_{p}$ in each configuration of system (A – F) demonstrate the changes that are obtained for each requirement of robustness and characteristics of the system and the need to select that configuration that guarantees, before the changes in scenarios, better performance in their indicators. An increment of the NPV of the network is related to the increment of the infrastructure investment level coinciding with the improvement of the robustness requirements of the solution. In other words, this increase in investment in network infrastructure generates significant robustness, reducing emissions and distances traveled by motorized vehicles.

The mathematical model implemented with the FePIA methodology has been validated to evaluate the different dynamic processes in a raw milk collection system. Thus, this study offers a comprehensive understanding of the configuration and evaluation of first-mile logistics systems focusing on the extended perspective of network behavior considering the behavior of system performance characteristics when disturbed. To evaluate the robust design of the logistics network, we studied the changes in the disturbance parameters that influence the topological structure of the network and its performance characteristics, determining the configuration in which, before these changes, the performance of the network logistics present fewer changes. Finally, this study enriches the emerging research on robust logistics system design that still needs to fully incorporate the role of various possible network configurations and their evaluations. We address this aspect by studying the dynamic nature of the raw milk collection logistics network (scenarios) interrelated with its robust design.

The numerical experiments showed that the implemented methodology serves to select a robust logistic system despite the change in the disturbing parameters of "normal" conditions. Despite changes in disturbing parameters for system performance, decision-makers can use the methodology to choose a system configuration adequate to manage disturbances. Furthermore, this study sheds light on how to manage first-mile logistics networks for perishable products.

## 5 Concluding remarks

This work has been motivated by the need for small dairy producers to generate strategies to guarantee the quality of the product from the beginning of the chain, meeting the requirements of the final customer demanded by dairy company processors. This work demonstrated the possibility of evaluating the robustness of a raw milk collection chain by using the FePIA methodology through a multi-objective analysis based on social, environmental, and economic aspects. Considering the effects on strategic and tactical decisions, farmers must compel those small producers to stay in the market to sell raw milk.

This research is limited and can be expanded in several ways. In this research, the attention was focused on the transportation and distribution of raw milk in small companies. At the same time, other perishable products can be addressed by modifying the optimization model and the robust design methodology of the proposed logistics system. The calculation of emissions from fuel use has been developed from a mathematical model that can be applied to other scenarios, different from the use of motorcycles for transporting perishable products. A limitation of the study is that the parameters of the raw milk collection of a supply chain may be uncertain. Therefore, these values can be considered for the future development of the model. Besides, Euclidean distance was used to calculate the distance between raw milk producers and tanks. Given the closeness between producers, this value is a good approach. However, the distance must be calculated with greater precision for logistics network designs at interregional scales.

The results show that the performance of the sustainable logistics system, based on financial, environmental, and social aspects, provides a more realistic analysis to support decision-making. From this analysis, it is concluded that the configuration of a sustainable logistics system for the company's formation is feasible. This conclusion is based explicitly on the high impacts of the analysis of CO_2_ emissions from transporting milk through motorcycles. Furthermore, it is essential to note that the solutions obtained by the present approach indicate that the model can provide Pareto curves to assess trade-offs between economic and environmental objectives. The decisions of location (strategic), use of transportation modes (tactical), use of cooling tank capacities (tactical), and distribution (operational) guarantee a better performance of the global system.

The multi-objective approach to this problem is appropriate since the balance between economic (NPV) and environmental (CO_2_ emissions) aspects can be selected from the Pareto efficient frontier through a decision-making process based on the methodology FePIA. One of the most interesting conclusions of this study is that using more motorcycles can generate excellent fuel economy in the long term and, therefore, fewer emissions and less use of human force in transporting raw milk canteens. From the point of view of environmental impact, more motorcycles that make short paths transporting raw milk from primary producers with more excellent supply are the appropriate strategy.

The results of this study show that dairy companies can make the best decisions for their first-mile logistics system for the storage, facility location, and refrigeration of raw milk, obtaining profitable solutions and guaranteeing good economic results, and reducing CO_2_ emissions. Finally, developing more efficient, robust supply methodologies is essential to obtain better financial and environmental results, guaranteeing better income in the long term. In addition, other transportation modes must be included, such as vans or small tank trucks. Future research can consider different types of uncertainty for the input parameters. Additionally, planning decisions for tactical and operational first-mile logistics systems can be approached under the sustainability paradigm by expanding the current approach. Likewise, the present work can deal with uncertainty for food supply chains. Finally, new robustness metrics can be tested to incorporate the study of the first-mile logistics system and the supply chain design.

Finally, although the proposed methodology is generic, the analysis results are limited to the time, location, and specific characteristics of the case studied and cannot be generalized to other cases. Therefore, a series of comprehensive experiments with other food product pickup systems may shed more light on the proposed methodology for the robust design of first-mile product pickup logistics systems and the results in future research. Future research must be addressed regarding the problems generating the disturbance of the supply chain and defining each possible scenario's occurrence probability. Besides, several objectives must be considered as the key performance of the supply chain, such as those proposed by Refs. [[111], [112], [113], [114], [115]]. Finally, methodologies to solve stochastic supply chain models could be considered, such as the Sample Average Approximation (SAA) ([[116], [117], [118], [119], [120]]) and the scenario-based method ([124]).

## Author contribution statement

Andrés Polo Roa: Conceived and designed the experiments; Performed the experiments; Analyzed and interpreted the data; Contributed reagents, materials, analysis tools or data; Wrote the paper.

John Willmer Escobar: Conceived and designed the experiments; Analyzed and interpreted the data; Contributed reagents, materials, analysis tools or data.

María Paula Montoya: Conceived and designed the experiments; Contributed reagents, materials, analysis tools or data; Wrote the paper.

## Data availability statement

Data will be made available on request.

## Declaration of competing interest

The authors declare that they have no known competing financial interests or personal relationships that could have appeared to influence the work reported in this paper.

## Figures and Tables

**Figure A1:**
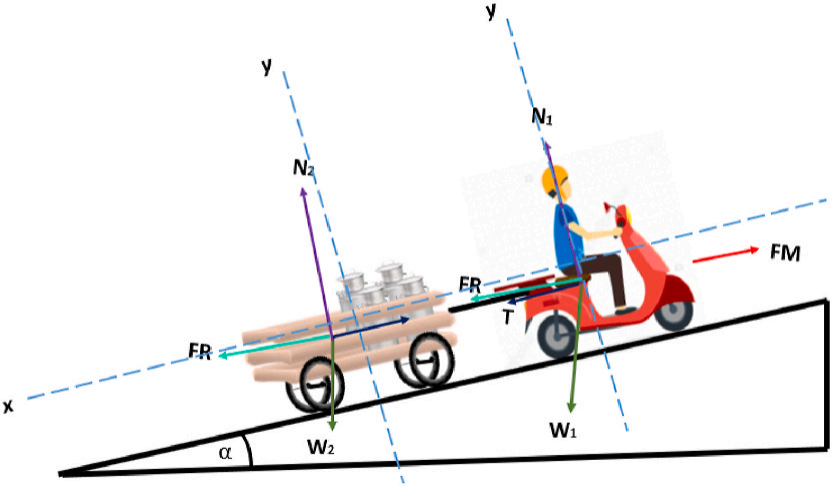
Producer going up.

**Figure A2:**
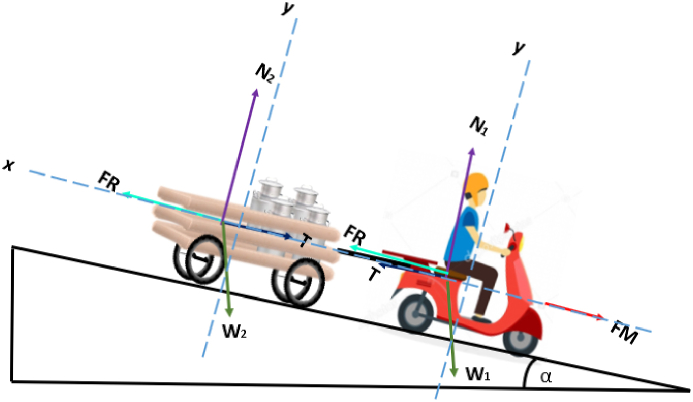
Producer going down.

**Graph 2:**
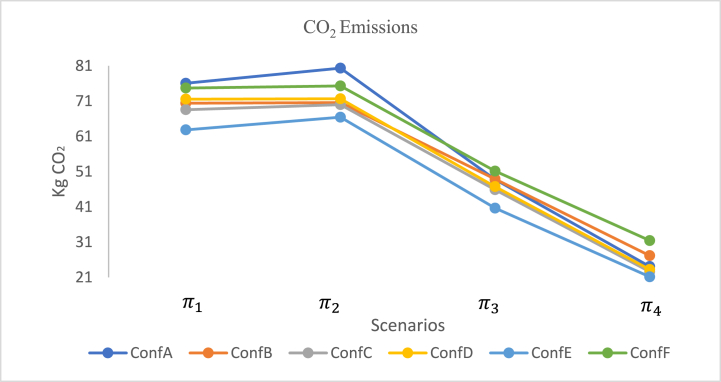
Obtained results for $\Gamma_{2}$.

**Figure A3:**
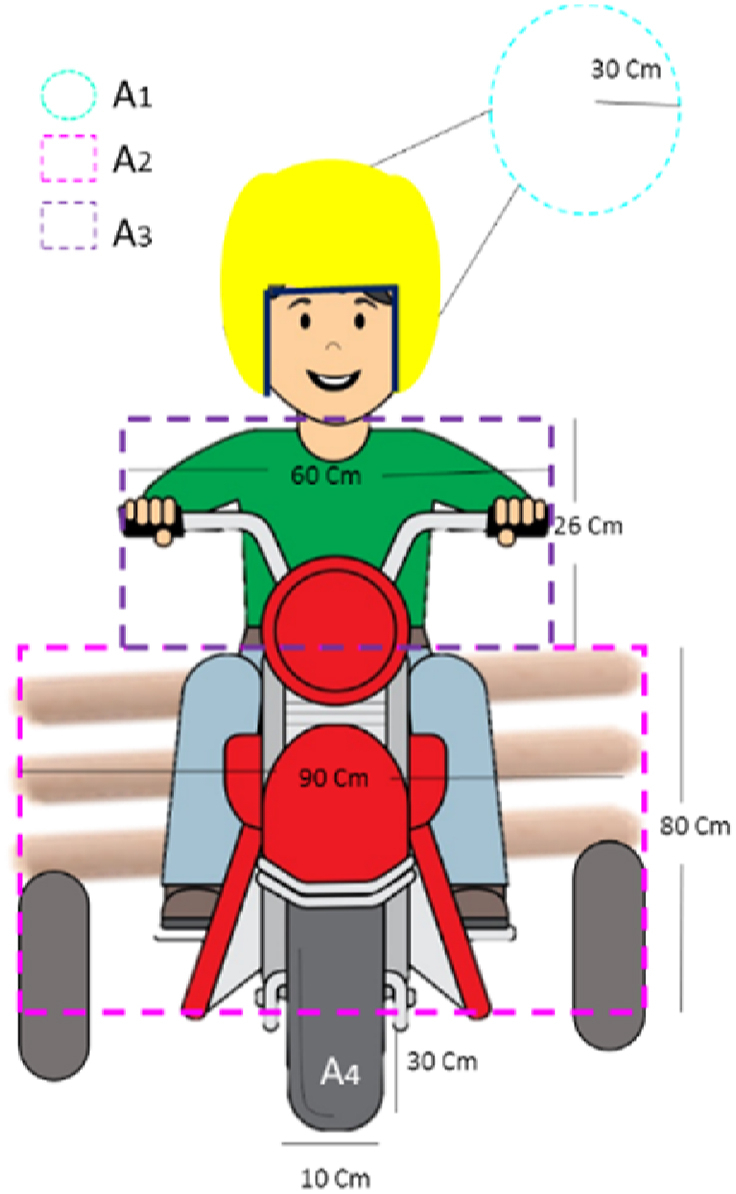
Diagram for calculation of wind friction.

**Graph 2:**
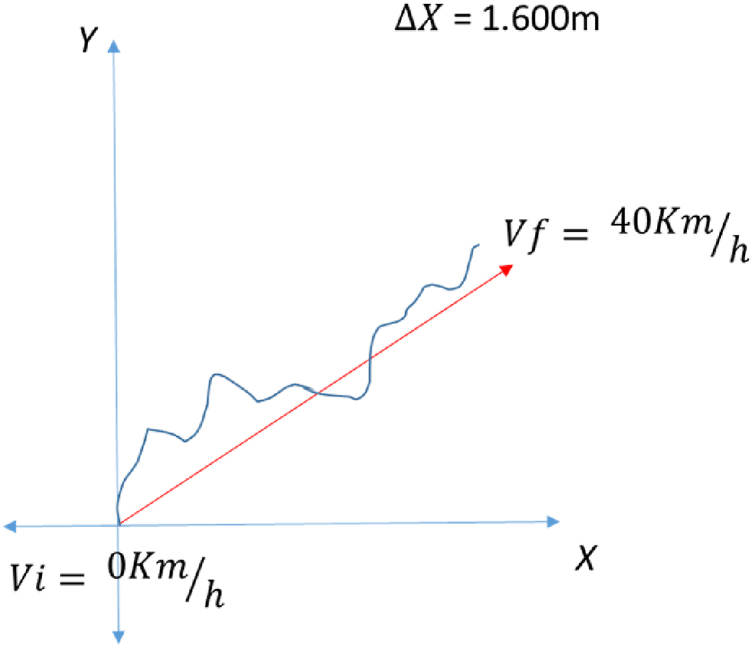
Diagram for calculation of acceleration.

**Table A1:** Information of cooling tanks

Type of tank	Investment (COP/tank)	Tank Capacity (lts/year)	Depreciation cost by tank (COP/Year)			Cooling cost (COP/lts)			Depreciation cost (COP/Year)		
			Year 1	Year 2	Year 3	Year 1	Year 2	Year 3	Year 1	Year 2	Year 3
t1	2.500.000	187.200	500.000	500.000	500.000	48.51	48.51	48.51	250.000	250.000	250.000
t2	2.900.000	234.000	590.000	590.000	590.000	39.08	39.08	39.08	290.000	290.000	290.000
t3	9.700.000	432.000	715.000	715.000	715.000	33.56	33.56	33.56	970.000	970.000	970.000
t4	1.1600.000	576.000	795.000	795.000	795.000	29.65	29.65	29.65	1.160.000	1.160.000	1.160.000
t5	1/5400.000	720.000	925.000	925.000	925.000	26.61	26.61	26.61	1.540.000	1.540.000	1.540.000

**Table A2:** Information of producers

Producer	Longitude (°)	Latitude (°)	Number of bovines
i1	73.789195	5.682394	5
i2	73.790912	5.687176	6
i3	73.786964	5.691105	12
i4	73.781471	5.689226	6
i5	73.777437	5.690678	5
i6	73.783531	5.68487	5
i7	73.785333	5.676159	7
i8	73.792285	5.675305	12
i9	73.799581	5.672486	9
i10	73.796834	5.67781	22
i11	73.800353	5.681796	8
i12	73.796062	5.683675	10
i13	73.794603	5.687945	5
i14	73.799323	5.687603	6
i15	73.803615	5.688287	12
i16	73.809966	5.679917	8
i17	73.810996	5.688628	20
i18	73.804731	5.691532	5
i19	73.784389	5.693411	8
i20	73.786535	5.698706	4
i21	73.793788	5.685639	11
i22	73.782329	5.694522	5
i23	73.785161	5.693667	15
i24	73.785333	5.698109	22
i25	73.787822	5.700073	15
i26	73.808336	5.681967	4
i27	73.804988	5.67966	10
i28	73.793058	5.695119	5
i29	73.810567	5.689055	12
i30	73.809194	5.682991	18

## Appendix

### Appendix-A1

The calculation of the associated expenses with the economic activity of the company, according to averages detected, are as follows:

Total sales per producer

(A-1) $${VenP}_{i,j}=\left(0.95*{Ofer}_{i,j}*{Prec}_{j}\right)\forall i=0,1,\ldots ,i;\forall j=0,1,\ldots ,J$$

Cow concentrate inventories

(A-2) $${{Conc}_{i,j}=VenP}_{i,j}*0.3\forall i=0,1,\ldots ,i;\forall j=0,1,\ldots ,J$$

Inventories of fertilizer for planting grass

(A-3) $${Fert}_{i,j}={VenP}_{i,j}*0.08\forall i=0,1,\ldots ,i;\forall j=0,1,\ldots ,J$$

Milk sales for period j,

(A-4) $$\sum_{\forall i}{VenP}_{i,j}\forall j=0,1,\ldots ,J$$

Value of initial inventory by producer i for period j,

(A-5) $${InvPr}_{i,j}={Conc}_{i,j}+{Fert}_{i,j}\forall i=0,1,\ldots ,i;\forall j=0,1,\ldots ,J$$

Value of final inventory by producer i for period j,

(A-6) $${InvFin}_{i,j}={InvPr}_{i,j}*0.07$$

### Appendix-A2

Work (W) to establish the vehicles' fuel consumption and total emission

In this study, the use of motorcycles by some of the producers is considered. These vehicles are associated with fuel consumption per km traveled. The fuel consumption is related to the work (W) performed by the motorcycle engine to complete routes and where (W) is affected by the forces acting on the producer driving the motorcycle and on the vehicle itself. We have considered two possibilities: going up and down by motorcycle on terrain with a slight incline, generating more Work (W). The possibilities are shown respectively in Figs. 6 and 7.

According to Figures A1 and A2, the calculation of the motor force (FM) is as follows ([121]):

(A-7) $$\sum F_{X1=}\;FM-w_{1}x-{Fr}_{1}-T=m_{1}*a$$

(A-8) $$\sum F_{X2=}\;T-w_{2}x-{Fr}_{2}=m_{2}*a$$

(A-9) $$\sum F_{Y1}=N_{1}-w_{1}y=0$$

(A-10) $$\sum F_{Y2=}N_{2}-w_{2}y=0$$

Where:

(A-11) $$FM=\left(m_{1}+m_{2}\right)*a+w_{1}x+w_{2}x+Fr$$

(A-12) $$w_{1}x=m_{1}g\;\cos\;\theta$$

(A-13) $$w_{2}x=m_{2}g\;\cos\;\theta$$

Considering the following aspects:

(A-14) Fr=μ*Nμ=0,7Dryroadby

Therefore, the value of Fr could be reduced for each case [122]

(A-15) $${Fr}_{1}=\mu *m_{1}\;g*sen\;\theta =>{Fr}_{1}=6,86*m_{1}*sen\;\theta$$

(A-16) $${Fr}_{2}=\mu *m_{2}\;g*sen\;\theta =>{Fr}_{2}=6,86*m_{1}*sen\;\theta$$

(A-17) $$Fr=-{Fr}_{1}+{Fr}_{2}+{Fr}_{wind}$$

For the calculation of ${Fr}_{wind}$, we have to consider the Figure A3 [122].

According to Figure A3, we are able to obtain the following data ([121]):

(A-18) $${Fr}_{wind}=0,2*\rho *A*V^{2}$$

(A-19) *A* *=* Area

(A-20) ρ=Airdensity=1,225Kgm3

(A-21) V=Speed=40Kmh

### Calculate driver head area

(A-22) $$A_{1}=\pi *r^{2}$$

(A-23) $$A_{1}=\pi *{\left(0,15m\right)}^{2}$$

(A-24) $$A_{1}=0,071m^{2}$$

Calculate driver's torso area:

(A-25) $$A_{2}=0,26*0,60$$

(A-26) $$A_{1}A_{2}=0,16m^{2}$$

Calculate cart area:

(A-27) $$A_{3}=80*90$$

(A-28) $$A_{3}=0,72m^{2}$$

Calculate tire area:

(A-29) $$A_{4}=30*10$$

(A-30) $$A_{4}=0,03m^{2}*3$$

(A-31) $$A_{4}=0,09\;m^{2}$$

(A-32) $$A\;Total=A_{1}+A_{2}+A_{3}+A_{4}$$

(A-33) $$A\;Total={0,071m}^{2}+{0,160m}^{2}+{0,720m}^{2}+{0,09m}^{2}$$

(A-34) $$A\;Total={1,041m}^{2}$$

With the ${Fr}_{wind}$ the Fr finally results in the following:

(A-35) $$Frair=0,2*\rho *A*V^{2}$$

(A-36) Frwind=0,2*1,225Kgm3*1,041m2*(40Kmh)ˆ2

(A-37) Frair=31,49N

(A-38) $$Fr=-\left(6,86\left(m_{1}sen\theta +m_{2}sen\theta \right)+31,49N\right)$$

(A-39) $$Fr=-\left(6,86*sen\theta \left(m_{1}+m_{2}\right)+31,49N\right)$$

The calculation of the acceleration a is carried out as follows:

Where:

(A-40) $${Vf}^{2}={Vi}^{2}+2a\;\left(\Delta X\right)$$

(A-41) $$\frac{{Vf}^{2}}{2\left(\Delta X\right)}=a=\frac{\left({40Km/h}^{2}\right)}{2\left(1,6km\right)}$$

(A-42) a=500Km/h

(A-43) $$a=500\frac{Km}{h}=0,0385\;m/s^{2}$$

Motor Force (FM) is calculated as follows:

Motorcycle going up:

(A-44) $$FM=\left(m_{1}+m_{2}\right)a+w_{1}x+w_{2}x+Fr$$

(A-45) $$FM=\left(m_{1}+m_{2}\right)\;a+\left(9,8\;sen\;\theta \left(m_{1}+m_{2}\right)\right)+\left(6,86*\cos\;\theta \left(m_{1}+m_{2}\right)+31,49N\right)$$

(A-46) $$FM=\left(m_{1}+m_{2}\right)\left(a+9,8\;sen\;\theta +6,86*\cos\;\theta +\frac{31,49N}{\left(m_{1}+m_{2}\right)}\right)$$

Motorcycle going down:

(A-47) $$FM=-a\left(m_{1}+m_{2}\right)+w_{1}x+w_{2}x+Fr$$

(A-48) $$Fm=\left(m_{1}+m_{2}\right)\left(6,86*9,8*\cos\;\theta \right)+\left(\frac{31,49N}{\left(m_{1}+m_{2}\right)}\right)-\left(9,8*sen\theta \right)-a$$

Work (W) establishes the vehicles' fuel consumption and total emission.

(A-49) W=Fm*Traveleddistance

The calculation of Fm involves the average weight of the motorcycle, the weight of the cart, the weight transported in milk, and the weight of the total number of used canteens. The equations are calculated either with the producer going up on the motorcycle with load (A), going down with a load (B), going up without load (C), and going down without load (D) for the 360 days of the year:

(A-50) $${CCSUB}_{i,m,j}=\left(\left(PMot*360\right)+\left(Pcar*360\right)+{Ptra}_{i,j}+\left(Pcant*{CANT}_{i,j}\right)\right)*{Moto}_{i}\;\left(A\right){CCSUB}_{i,m,j}\;Parameter\;representing\;the\;producer\;on\;the\;motorcycle\;going\;up\;with\;load$$

(A-51) $${CCBAJ}_{i,m,j}=\left(\left(PMot*360\right)+\left(Pcar*360\right)+{Ptra}_{i,j}+\left(Pcant*{CANT}_{i,j}\right)\right)*{Moto}_{i}\;\left(B\right){CCBAJ}_{i,m,j}\;Parameter\;representing\;the\;producer\;on\;the\;motorcycle\;going\;down\;with\;load$$

(A-52) $${SCSUB}_{i,m,j}=\left(\left(PMoto*360\right)+\left(Pcar*360\right)+\left(Pcant*{CANT}_{i,j}\right)\right)*{Moto}_{i}\;\left(C\right){SCSUB}_{i,m,j}\;Parameter\;representing\;the\;producer\;on\;the\;motorcycle\;going\;up\;without\;load$$

(A-53) $${SCBAJ}_{i,m,j}=\left(\left(PMoto*360\right)+\left(Pcar*360\right)+\left(Pcant*{CANT}_{i,j}\right)\right)*{Moto}_{i}\;\left(D\right){SCBAJ}_{i,m,j}\;Parameter\;representing\;the\;producer\;on\;the\;motorcycle\;going\;down\;without\;load$$

Calculating the motor work W for each option (A-50) – (A-53) is possible. In particular, the different forces associated with fuel consumption are calculated concerning the work of the motorcycle engine with load, without load, going up or down.

(A-54) $${W1}_{i,m,t,}=\left({CCSUB}_{i,m,j}*\left(\left(ace+\left(Gra*sen\;\left(B_{i,m}\right)\right)+\left(\left(Coef*Gra\right)*\cos\left(B_{i,m}\right)\right)+(\frac{Fraire}{{CCSUB}_{i,m,j}}\right)\right)\right)*{BBIN}_{i,m}{W1}_{i,m,t,}\;Motorcycle\;force\;going\;up\;with\;load$$

(A-55) $${W2}_{i,m,t,}=\left({SCSUB}_{i,m,j}*\left(\left(ace+\left(Gra*sen\;\left(B_{i,m}\right)\right)+\left(\left(Coef*Gra\right)*\cos\left(B_{i,m}\right)\right)+(\frac{Fraire}{{SCSUB}_{i,m,j}}\right)\right)\right)*\left(1-{BBIN}_{i,m}\right){W2}_{i,m,t,}\;Motorcycle\;force\;going\;up\;without\;load$$

(A-56) $${W3}_{i,m,t,}=\left({CCBAJ}_{i,m,j}*\left(\left(Coef*Gra\right)*\cos\left(B_{i,m}\right)\right)+\left(\frac{Fraire}{{CCBAJ}_{i,m,j}}\right)-\left((Gra*sen\;\left(B_{i,m}\right)\right)-ace\right))*\left(1-{BBIN}_{i,m}\right)){W3}_{i,m,t,}\;Motorcycle\;force\;going\;down\;with\;load$$

(A-57) $${W4}_{i,m,t,}=\left({SCBAJ}_{i,m,j}*\left(\left(Coef*Gra\right)*\cos\left(B_{i,m}\right)\right)+\left(\frac{Fraire}{{SCBAJ}_{i,m,j}}\right)-\left((Gra*sen\;\left(B_{i,m}\right)\right)-ace\right))*\left({BBIN}_{i,m}\right)){W4}_{i,m,t,}\;Motorcycle\;force\;going\;down\;without\;load$$
